# Role of ergosterol biosynthesis in growth, drug sensitivity, and host colonization of honey bee trypanosomatid parasite, *Lotmaria passim*

**DOI:** 10.1093/femsmc/xtag020

**Published:** 2026-04-15

**Authors:** Xuye Yuan, Tatsuhiko Kadowaki

**Affiliations:** Department of Biosciences and Bioinformatics, School of Science, Xi’an Jiaotong-Liverpool University, Suzhou Dushu Lake Higher Education Town, Jiangsu Province 215123, China; Department of Biosciences and Bioinformatics, School of Science, Xi’an Jiaotong-Liverpool University, Suzhou Dushu Lake Higher Education Town, Jiangsu Province 215123, China

**Keywords:** honey bee trypanosomatid parasite, *Lotmaria passim*, ergosterol biosynthesis, gene disruption by CRISPR

## Abstract

*Lotmaria passim* is a widespread trypanosomatid parasite of the honey bee gut, yet the metabolic pathways underpinning its growth and infection remain poorly defined. Like other kinetoplastids, *L. passim* synthesizes ergosterol as its principal membrane sterol, but the functional significance of this pathway in an insect-associated lineage has not been experimentally assessed. We investigated ergosterol biosynthesis in *L. passim* through phylogenetic analyses, targeted gene disruption, sterol profiling, and honey bee infection assays. Early sterol biosynthetic enzymes displayed non-canonical evolutionary affiliations, whereas late stage enzymes localized to the endoplasmic reticulum. Genetic analyses showed that sterol C-8 isomerase appears to be essential for parasite viability, while sterol C-5 desaturase (SC5D) is dispensable *in vitro. SC5D*-null mutants (*Lp*Δ*SC5D*) accumulated ergosta-7,22-dienol, confirming its role in the final step of ergosterol synthesis. Although *Lp*Δ*SC5D* grew normally at optimal temperature, they exhibited temperature-sensitive growth defects, abnormal morphology, amphotericin B resistance, and a marked reduction in honey bee gut colonization. These findings demonstrate that while *L. passim* can tolerate altered sterol composition in culture, intact ergosterol biosynthesis is critical for environmental resilience and successful host infection. Sterol metabolism thus emerges as a key determinant of fitness and infection in this insect–associated trypanosomatid.

## Teaser

Ergosterol is the major membrane sterol in *Lotmaria passim* and required for efficient colonization of the honey bee gut.

## Introduction

Trypanosomatid parasites comprise a diverse group of flagellated protists that occupy a wide range of ecological niches and host environments. While several species, such as *Leishmania* and *Trypanosoma*, are well known for their medical and veterinary importance, others have more recently emerged as parasites of insects, including pollinators. *Lotmaria passim* is a monoxenous trypanosomatid that colonizes the gut of the honey bee (*Apis mellifera*) and is now recognized as one of the most prevalent trypanosomatid parasites of managed and wild bee populations (Tiritelli et al. 2025). Despite its broad distribution and potential impact on host health, the basic cell biology and metabolic requirements that underlie *L. passim* survival and infection remain poorly characterized.

Sterols are essential components of eukaryotic membranes, where they regulate membrane fluidity, permeability, and the function of embedded proteins. Most eukaryotes synthesize characteristic sterol end products: animals primarily produce cholesterol, plants produce phytosterols, and fungi synthesize ergosterol (Ferreira-Guerra et al. 2025). Kinetoplastids, including *L. passim*, share the fungal-type pathway and use ergosterol and related sterols as their major membrane components (Goad et al. 1984, Roberts et al. 2003). In pathogenic *Leishmania* species, ergosterol biosynthesis has been intensively studied, in part because several pathway enzymes are targets of clinically important drugs such as azoles and polyenes (Goldsmith and Perry 2004). In contrast, much less is known about the role of sterol metabolism in monoxenous trypanosomatids and, in particular, how sterol composition influences parasite fitness in insect hosts.

The ergosterol biosynthesis pathway consists of a series of enzymatic reactions that are spatially partitioned within the cell and tightly regulated. In trypanosomatids, early steps of the pathway occur in the glycosome, whereas later reactions are associated with the endoplasmic reticulum (Opperdoes and Szikora 2006, Xu et al. 2014). Genetic and pharmacological studies in *Leishmania* spp. have revealed that disruption of sterol synthesis can have diverse consequences, ranging from subtle growth defects to severe impairments in differentiation, morphology, and drug sensitivity (Mwenechanya et al. 2017, Mukherjee et al. 2019, Pountain et al. 2019, Ning et al. 2020, Alpizar-Sosa et al. 2022, Jin et al. 2024, Ning et al. 2024, Tulloch et al. 2024). Notably, some late-stage enzymes are dispensable for parasite survival under culture conditions, suggesting that certain sterol intermediates can partially compensate for the absence of ergosterol. Whether such flexibility extends to other trypanosomatids, and whether it is compatible with successful infection of insect hosts, remains an open question.

Recent comparative genomic analyses have further suggested that the sterol biosynthesis pathway in kinetoplastids has a complex evolutionary history, with several enzymes showing affinities to non-eukaryotic homologs(Opperdoes and Szikora 2006, Opperdoes et al. 2016). Although these observations raise intriguing questions about pathway evolution, the functional significance of sterol biosynthesis for parasite biology is ultimately defined by its contribution to cellular physiology and host interaction. In the case of *L. passim*, no experimental data have been available to link sterol metabolism to growth, stress tolerance, or colonization of the honey bee gut.

In this study, we combine phylogenetic analysis, targeted gene disruption, biochemical profiling, and infection experiments to investigate the role of ergosterol biosynthesis in *L. passim*. Focusing on late-stage enzymes, we examine the essentiality and functional specialization of sterol C-8 isomerase (SC8I) and sterol C-5 desaturase (SC5D), assess the consequences of altered sterol composition for parasite growth, morphology, and drug sensitivity, and directly test the requirement for ergosterol during infection of the honey bee host. Our results reveal that, while *L. passim* can tolerate substantial changes in sterol composition *in vitro*, ergosterol is indispensable for efficient host colonization, highlighting sterol metabolism as a central determinant of parasite fitness in its natural ecological context.

## Materials and methods

### Culture of *L. passim*

*Lotmaria passim* promastigotes (PRA-403) was obtained from the American Type Culture Collection and cultured in the modified FP-FB medium (Salathé et al. 2012) at 25°C without CO_2_.

### Identification of *L. passim* genes involved in ergosterol biosynthesis

Since no public annotation existed for *L. passim* genomes (strain 422; GCA_034478905.1) (Markowitz et al. 2025, Ruiz et al. 2025), a new genome annotation was generated using the BRAKER pipeline (Lomsadze et al. 2005, Stanke et al. 2006, Gotoh 2008, Stanke et al. 2008, Iwata and Gotoh 2012, Buchfink et al. 2015, Hoff et al. 2016, Brůna et al. 2020, 2021, Gabriel et al. 2021). Gene prediction was performed using GeneMark–EP with default parameters and the –gff3 output option, incorporating *Crithidia bombi, Leptomonas pyrrhocoris*, and *Leptomonas seymouri* as reference species. Annotation quality was evaluated with AGAT v1.5.1, which identified 8119 predicted protein–coding genes with an average CDS length of 2340 bp (∼780 amino acids). BUSCO v6.0.0 (euglenozoa_odb12 dataset, 3611 markers) (Manni et al. 2021) further confirmed the robustness of the annotation, showing 90.7% completeness (87.2% single–copy and 3.5% duplicated), 0.8% fragmented, and 8.5% missing genes.

To identify genes involved in ergosterol biosynthesis, we performed BLAST searches using *Saccharomyces cerevisiae* ERG genes as queries. Orthologous sequences from *L. passim* were identified in the above annotated proteins. Other kinetoplastid sequences were retrieved from the TriTrypDB database (Shanmugasundram et al. 2023).

### Phylogenetic analysis of genes involved in ergosterol biosynthetic pathway

For each target gene, the top 250 homologs were identified via an online NCBI BLASTP search against the non-redundant (nr) database, with all other parameters set to default. When top hits were bacterial or archaeal proteins, homologs from diverse eukaryotic lineages—*Homo sapiens* (Animalia/Chordata), *Branchiostoma floridae* (Animalia/Chordata), *Nematostella vectensis* (Animalia/Cnidaria), *Arabidopsis thaliana* (Plantae/Magnoliophyta), *Oryza sativa* (Plantae/Magnoliophyta), *Chlamydomonas reinhardtii* (Viridiplantae/Chlorophyta), *Dictyostellum discoideum* (Amoebozoa/Dictyostelia), *Acanthamoeba castellanii* (Amoebozoa/Discosea)*, Guillardia theta* (Chromista/Cryptophyta), *Saccharomyces cerevisiae* (Fungi/Ascomycota), *Agaricus bisporus* (Fungi/Basidiomycota), *Emiliania huxleyi* (Chromista/Cryptophyta), *Tetrahymena thermophila* (Chromista/Ciliophora), and *Phytophthora infestans* (Chromista/Oomycota)—were retrieved. These species represent Opisthokonta, Amoebozoa, Excavata, SAR, and Archaeplastida (Keeling and Burki 2019). Since Mevalonate kinase is absent in *C. reinhardtii, Chara braunii* (Viridiplantae/Charophyta) was used. The orthologs of *Bodo saltans, Leishmania major*, and *Trypanosoma cruzi* were identified from the TriTrypDB database as above. Multiple sequence alignments were performed using MUSCLE v5 (Edgar 2022), followed by trimming poorly aligned regions with trimAl (Capella-Gutiérrez et al. 2009) to improve alignment quality. Processed alignments were used to construct maximum likelihood (ML) trees using IQ-TREE2 (Minh et al. 2020), with optimal substitution models automatically selected. One thousand bootstrap replicates were performed to assess nodal support, and resulting trees were visualized using iTOL.

### Cellular localization of 3c-Myc-LpBiP, LpSC8I-GFP, LpSC5D1-GFP, and LpSC5D2-GFP in *L. passim*

To express triple c-Myc-tagged LpBiP (3c-Myc-LpBiP), the full open reading frame (ORF) of *LpBiP* (Binding Immunoglobulin Protein) was amplified by PCR using KOD-FX DNA polymerase (TOYOBO), *L. passim* genomic DNA, and primers 5-XhoI-LpBiP/3-XhoI-stop-LpBiP. The PCR product was digested with XhoI and cloned into a vector carrying triple c-Myc epitopes (Yuan and Kadowaki 2025), digested with the same enzyme. To construct LpSC8I-, LpSC5D1-, and LpSC5D2-GFP vectors, the ORFs were amplified using primer pairs LpSC8I-5-XbaI/LpSC8I-3-XbaI, LpSC5D1-5-XbaI/LpSC5D-3-XbaI, and LpSC5D2-5-XbaI/LpSC5D-3-XbaI. PCR products were digested with XbaI and cloned into the XbaI site of pTrex-n-eGFP plasmid (Peng et al. 2014) (Addgene #62 544). All sequences are listed in Supplementary Dataset 1. Actively growing *L. passim* cells (4 × 10^7^) were washed twice with PBS and resuspended in 0.4 ml Cytomix buffer (without EDTA) containing 20 mM KCl, 0.15 mM CaCl₂, 10 mM K₂HPO₄, 25 mM HEPES, and 5 mM MgCl₂ (pH 7.6). Electroporation was performed twice at 1-min intervals with 10 μg plasmid DNA using a Gene Pulser X cell electroporator (Bio-Rad) with a 2-mm gap cuvette, voltage 1.5 kV, capacitance 25 μF, and infinite resistance. Transfected parasites were cultured in 4 ml modified FPFB medium (Salathé et al. 2012), and blasticidin (50 μg/ml) and G418 (200 μg/ml) were added after 24 h to select drug-resistant clones.

Immunofluorescence detection was performed by washing and mounting parasites on poly-L-lysine-coated eight-well chamber slides, fixing with 4% paraformaldehyde, permeabilizing with 0.1% Triton X-100 in PBS (PT), and blocking with PT containing 5% normal goat serum (PTG). Samples were incubated overnight at 4°C with rabbit anti-GFP polyclonal antibody (1:1000, Proteintech) and CoraLite594-conjugated Myc tag monoclonal antibody (1:500, Proteintech) in PTG. After five washes with PT, samples were incubated with FITC-conjugated anti-rabbit IgG (Beyotime) for 2 h at room temperature, washed, stained with DAPI (1 µg/ml) for 5 min, and observed under a Nikon Eclipse Ni-U fluorescence microscope with 200 ms exposure.

### Disruption of *LpSC8I* and *LpSC5D* genes by CRISPR

The gRNA sequences for *LpSC8I* and *LpSC5D* were designed using a custom gRNA design tool (http://grna.ctegd.uga.edu (Peng and Tarleton 2015). The gRNAs with the highest overall scores were selected based on predicted on–target activity, potential off–target effects, nucleotide composition–derived efficiency, and the presence of bracketing microhomology pairs, including both homology length and proximity to the gRNA site. Complementary oligonucleotides for sgRNA sequences (LpSC8IgRNA378F/R and LpSC5DgRNA688RF/RR) were phosphorylated by T4 polynucleotide kinase (TAKARA), annealed, and cloned into BbsI-digested pSPneogRNAH vector (Zhang and Matlashewski 2015) (ADDGENE: # 63 556). The LpSC5D gRNA targets both *LpSC5D1* and *LpSC5D2. Lotmaria passim* expressing Cas9 (Liu et al. 2019) was electroporated with 10 μg plasmid DNA, and transformants were selected with blasticidin and G418. Donor DNA for *LpSC8I* consisted of 5’UTR (LpSC8I-5’UTR-F and LpSC8I-5’UTR-R), *Hph* ORF (LpSC8I-Hph-F and LpSC8I-Hph-R), and 3' UTR (LpSC8I-3'UTR-F and LpSC8I-3'UTR-R) fragments. They were fused and cloned into the EcoRV site of pBluescript II SK(+). For *LpSC5D* genes, 5'UTR of *LpSC5D1, Hph* ORF, and 3'UTR of *LpSC5D2* were fused to replace ORFs of both *LpSC5D1* and *LpSC5D2* by *Hph*. The linearized plasmid DNA (20 μg) digested with EcoRI (for *LpSC8I* donor DNA) or BamHI (for *LpSC5D* donor DNA) was used for electroporation of *L. passim* expressing both Cas9 and *LpSC8I* or *LpSC5D* gRNA, as described above. *Lotmaria passim* resistant to blasticidin, G418, and hygromycin was selected and cloned by serial dilutions in a 96-well plate. The genotype of each clone was first determined by the detection of 5’ wild-type (WT) and deleted (KO) alleles for *LpSC8I* or *LpSC5D1* by PCR. After identifying heterozygous (±) and homozygous (-/-) KO clones of *LpSC5D*, their 5’WT (LpSC5D1-5’UTR-Up and LpSC5D1-63R), 5’KO (LpSC5D1-5’UTR-Up and Hyg-159R), 3’WT (LpSC5D2-1782F and LpSC5D2-3'UTR-Down), and 3’KO (Hyg-846F and LpSC5D2-3'UTR-Down) alleles were confirmed by PCR. For *LpSC8I*, only heterozygous KO clones were identified by testing the 5'WT (LpSC8I-5'UTR-Up and LpSC8I-32R) and KO alleles (LpSC8I-5'UTR-Up and Hyg-159R).

Plasmid DNA expressing LpSC5D1 or LpSC5D2 was constructed by amplifying ORFs, using LpSC5D1-5-XbaI and LpSC5D-stop-HindIII or LpSC5D2-5-XbaI and LpSC5D-stop-HindIII primers. The plasmid DNA to express the corresponding GFP fusion protein was used as a template. The resulting PCR product was digested with XbaI and HindIII followed by subcloning into the same restriction enzyme sites of the tdTomato/pTREX-b plasmid DNA (Lander et al. 2015) (ADDGENE: #68 709) with bleomycin resistance gene. *LpSC5D*-deficient parasites (*Lp*Δ*SC5D*) were electroporated with above plasmid DNA and the parasites expressing LpSC5D1 (*Lp*Δ*SC5D*+LpSC5D1) or LpSC5D2 (*Lp*Δ*SC5D*+LpSC5D2) were selected with hygromycin and zeocin.

### RT-PCR

Total RNA from WT, *LpSC5D* heterozygous, and homozygous mutants was extracted using TRIzol (Sigma–Aldrich). Reverse transcription of 0.2 μg RNA was performed with ReverTra Ace (TOYOBO) and random primers, followed by PCR with GoTaq Green Master Mix (Promega). Both *LpSC5D1* and *LpSC5D2* mRNAs were detected using nested PCR, with primers LpSL-F-1st/LpSC5D-250R for first round and LpSL-F-2nd/LpSC5D1-63R for second round. Two reverse primers are complementary to both *LpSC5D1* and *LpSC5D2* mRNAs. *LpGAPDH* mRNA was detected with LpSL-F and LpGAPDH-R primers.

### Culture and cell body length measurement of *L. passim*

WT, *Lp*Δ*SC5D Lp*Δ*SC5D*+LpSC5D1, and *Lp*Δ*SC5D*+LpSC5D2 parasites were inoculated into the culture medium at 10^4^ cells/ml at 30°C. Cell counts were performed daily for 4 days using a hemocytometer. AmB was added at 0.1 µg/ml when necessary. For growth at 20°C, parasites were inoculated at 10⁵ cells/ml and counted for 5 days. The distance between the anterior and posterior end of cell body was measured at day 4 using Image-J. Two independent *Lp*Δ*SC5D* clones (A1 and C2) were phenotypically identical; C2 was used for further experiments.

### RT-qPCR

*LpSC5D*+LpSC5D1 and *Lp*Δ*SC5D*+LpSC5D2 parasites were cultured as described above, with five wells per genotype in a 24-well plate at 30 °C, and harvested on day 4. AmB was added to the culture medium as indicated. Total RNA was independently extracted from each well, treated with RNase-free DNase I (Promega), and reverse–transcribed in a 20 µl reaction as described above. The resulting cDNA was diluted three-fold with water, and *LpSC5D1* and *LpSC5D2* mRNA levels were quantified by qPCR using primers LpSC5D-607F and LpSC5D-709R. *Lotmaria passim* 18S rRNA served as the reference gene, amplified with primers Lp18S-F and Lp18S-R (Liu et al. 2020). Relative mRNA abundance was calculated using the ΔCt method, with one untreated *Lp*Δ*SC5D*+LpSC5D1 or *Lp*Δ*SC5D* +LpSC5D2 sample set to 1. For direct comparison of expression between *Lp*Δ*SC5D*+LpSC5D1 and *Lp*Δ*SC5D*+LpSC5D2, one *Lp*Δ*SC5D*+LpSC5D1 sample was used as the calibrator. Statistical significance was assessed using the Mann–Whitney test.

### GC-MS analysis of sterol in *L. passim*

Parasites were cultured to 10^7^ cells/ml in triplicate with 10 ml culture medium. Zeocin (50 µg/ml) was added to maintain plasmids in *Lp*Δ*SC5D*+LpSC5D1 and *Lp*Δ*SC5D*+LpSC5D2 parasites. Cells were collected, washed with 5 ml PBS, and extracted with 3 ml chloroform: methanol (2:1), with 20 µg cholesta-3,5-diene as internal control. 0.6 ml of 0.9% NaCl was added to separate phases; the lower phase was dried and suspended in 0.3 ml methanol. Electron impact GC/MS analysis of sterol was carried out using a Thermo Scientific ISQ single-stage quadrupole mass spectrometer coupled with a Trace GC system, operated through Thermo Xcalibur 2.1 software. Extracts (1 ml) were injected in splitless mode and separated on a Phenomenex ZB-50 column (15 m × 0.32 mm i.d., 0.5 µm film thickness). The GC oven was programmed as follows: an initial temperature of 100°C held for 2 min, ramped to 200°C at 50°C/min, then increased to 300°C at 10°C/min, with a final hold at 300°C for 10 min. The injector and transfer line temperatures were maintained at 280°C, and the ion source at 220°C. Mass spectra were acquired in full-scan mode (m/z 50–500) or as total ion current chromatograms, at a rate of one scan every 0.2 s. Electron ionization was performed at 70 eV. Mass specta of three focused peaks, cholesta–3,5–diene (used as an internal control), ergosterol, and ergosta–7,22–dienol are shown in Supplementary Dataset 2.

### Honey bee infection

Log-phase parasites (5 × 10⁵ cells/ml) were washed and suspended in 10% sucrose/PBS at 5 × 10^4^ cells/μl. Newly emerged honey bees were starved for 2–3 h, then 20 bees were fed 2 μl solution containing either WT, *Lp*Δ*SC5D, Lp*Δ*SC5D*+LpSC5D1, or *Lp*Δ*SC5D*+LpSC5D2 (10⁵ cells in total). WT-infected bees were fed 50% sucrose or sucrose containing 0.1 or 1 μg/ml AmB. Bees were maintained at 33°C for 14 days and frozen at −80°C. Four bees from each of three repeats (12 total per group) were analyzed. Genomic DNA from the whole abdomen of individual bee was extracted using DNAzol (Thermo-Fisher). *Lotmaria passim* abundance was quantified by qPCR with LpITS2-F/R primers targeting the internal transcribed spacer region 2 in the ribosomal RNA gene; honey bee AmHsTRPA was the reference gene with AmHsTRPA-F/R primers (Liu et al. 2020). Relative abundance was calculated using the ΔCt method, setting one WT sample fed 50% sucrose as 1. Statistical analysis was conducted with the Brunner-Munzel test. The amplification curves for qPCR are shown in Supplementary Dataset 3. All primer sequences are listed in Supplementary Dataset 4.

## Results

### Potential horizontal acquisition of key enzymes in the ergosterol biosynthesis pathway

**Figure 1:**
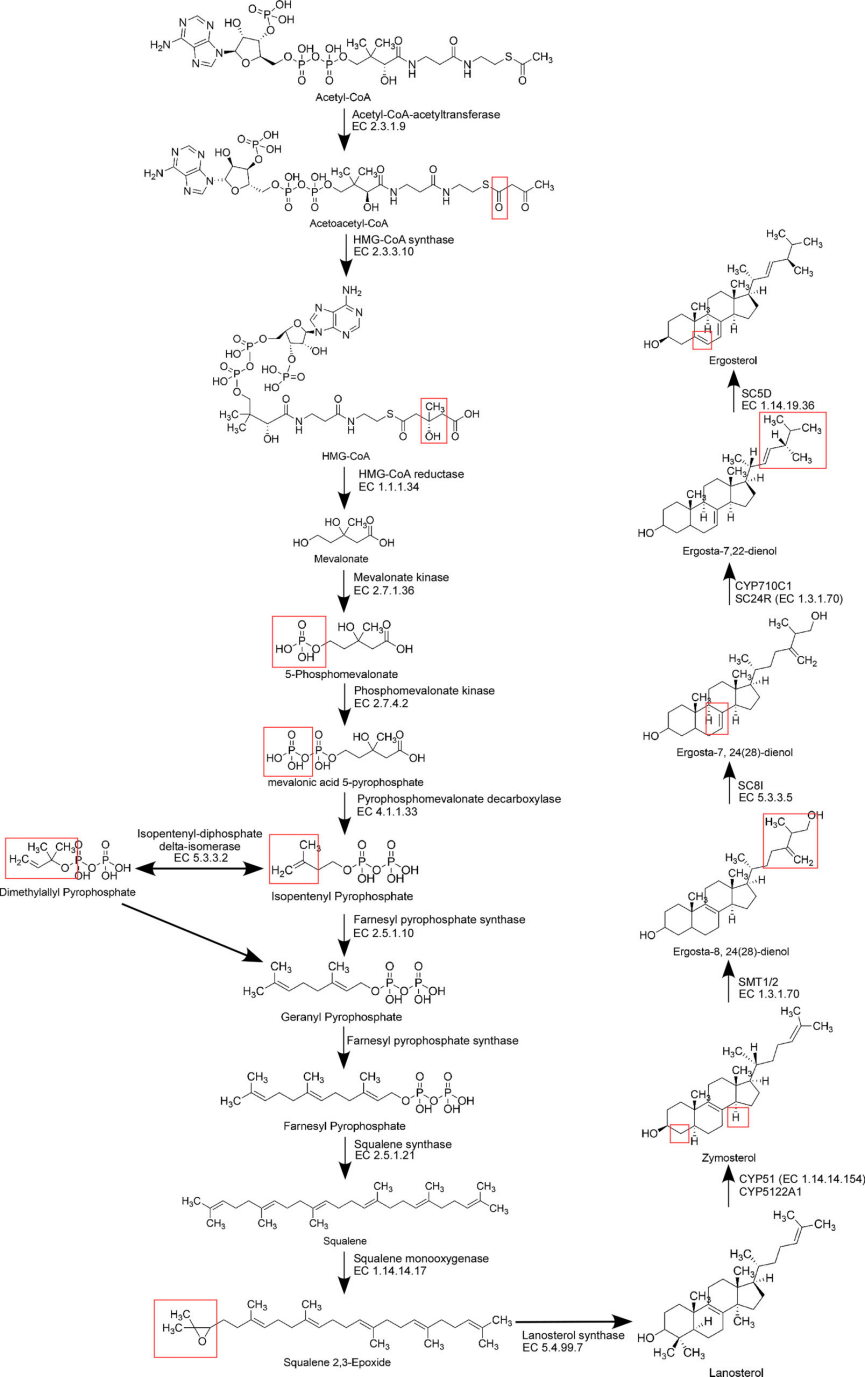
Ergosterol biosynthesis pathway. The pathway illustrates the metabolic steps from acetyl-CoA to ergosterol in trypanosomatids, along with the corresponding enzymes with EC numbers catalyzing each step. Subtle structural modifcations are highlighted in red. The pathway may branch from zymosterol if the enzymes act in a different order.

**Figure 2:**
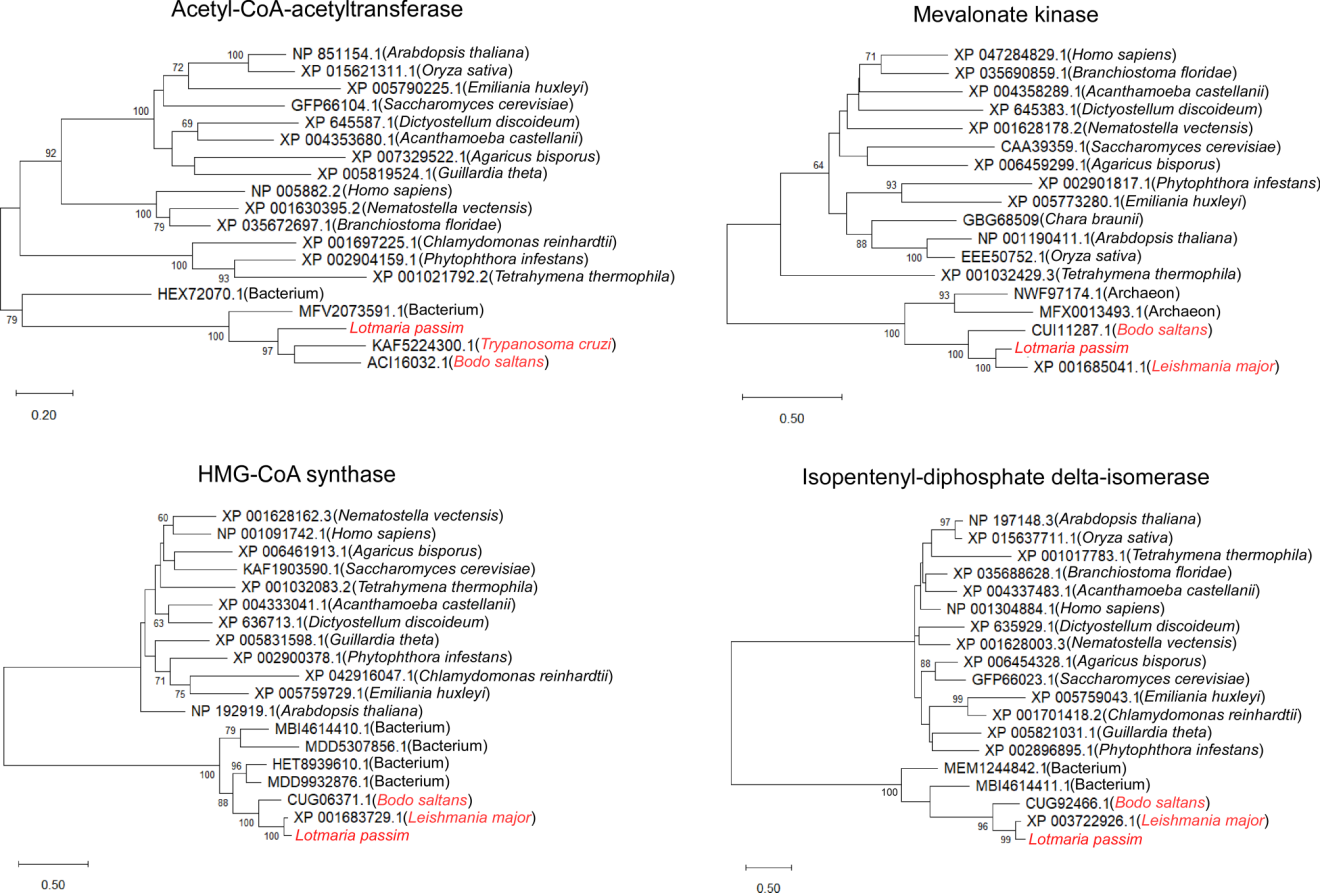
Evolutionary analysis of four HGT candidates using the maximum likelihood method. Phylogenetic trees were reconstructed for Acetyl-CoA acetyltransferase, HMG-CoA synthase, Mevalonate kinase, and Isopentenyl-diphosphate delta-isomerase from *L. passim, Leishmania major* (*Trypanosoma cruzi* for Acetyl-CoA acetyltransferase*), Bodo saltans*, various eukaryotes, bacteria, and archaea using the maximum likelihood method. Protein sequences are labeled with accession numbers and species names. Bootstrap values above 60 are shown at the nodes. Horizontal bars indicate 0.2–1 substitutions per site, depending on the protein.

Unlike animals and plants, which produce cholesterol or phytosterols, kinetoplastids and fungi synthesize ergosterol (Fig. 1). Early steps in sterol biosynthesis are conserved with bacteria, raising the possibility of bacterial origins. Phylogenetic analyses revealed that acetyl-CoA acetyltransferase, HMG-CoA synthase, and isopentenyl-diphosphate delta-isomerase from *L. passim, L. major* (or *T. cruzi*), and *B. saltans* cluster with bacterial proteins, while mevalonate kinase groups with archaeal homologs (Fig. 2). These results suggest that multiple early enzymes in the sterol biosynthesis pathway were acquired by horizontal gene transfer (HGT) in the kinetoplastid ancestor, then recruited to glycosomes in trypanosomatid parasites. This is consistent with prediction of glycosomal localization of mevalonate kinase and isopentenyl-diphosphate delta-isomerase in *L. major* (Opperdoes and Szikora 2006). In contrast, kinetoplastid HMG-CoA reductase, phosphomevalonate kinase, pyrophosphomevalonate decarboxylase, and farnesylpyrophosphate synthase do not cluster with bacterial homologs (Supplementary Dataset 5).

### Essentiality of sterol C-8 isomerase but not C-5 desaturase in *L. passim*

**Figure 3:**
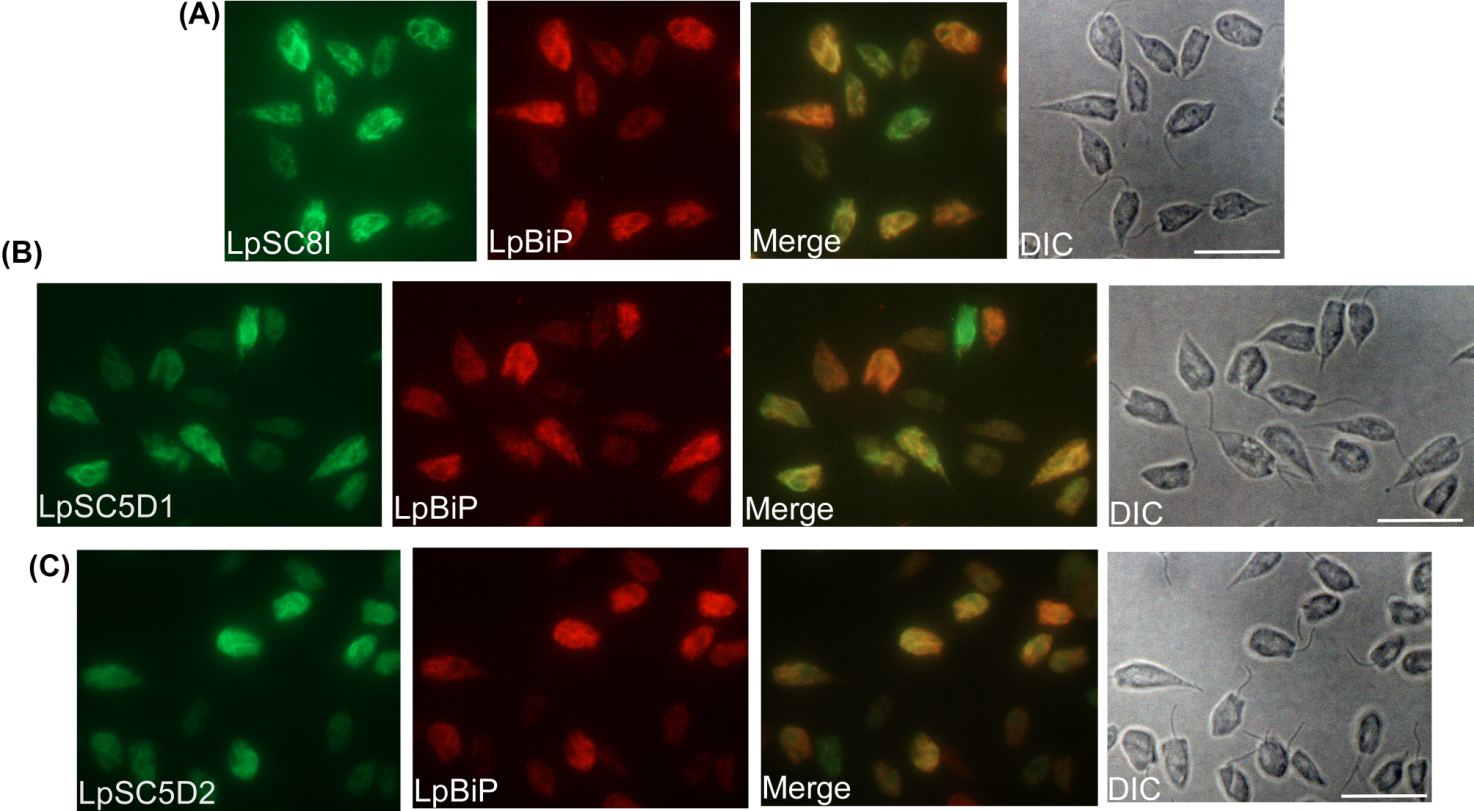
Intracellular localization of LpSC8I-, LpSC5D1-, and LpSC5D2-GFP in *Lotmaria passim. Lotmaria passim* expressing 3c-Myc-LpBiP (an ER marker) together with LpSC8I (A)-, LpSC5D1 (B)-, or LpSC5D2 (C)-GFP was examined by immunofluorescence and differential interference contrast (DIC) microscopy. Merged images of c-Myc and GFP signals are also shown. Scale bar: 10 μm.

**Figure 4:**
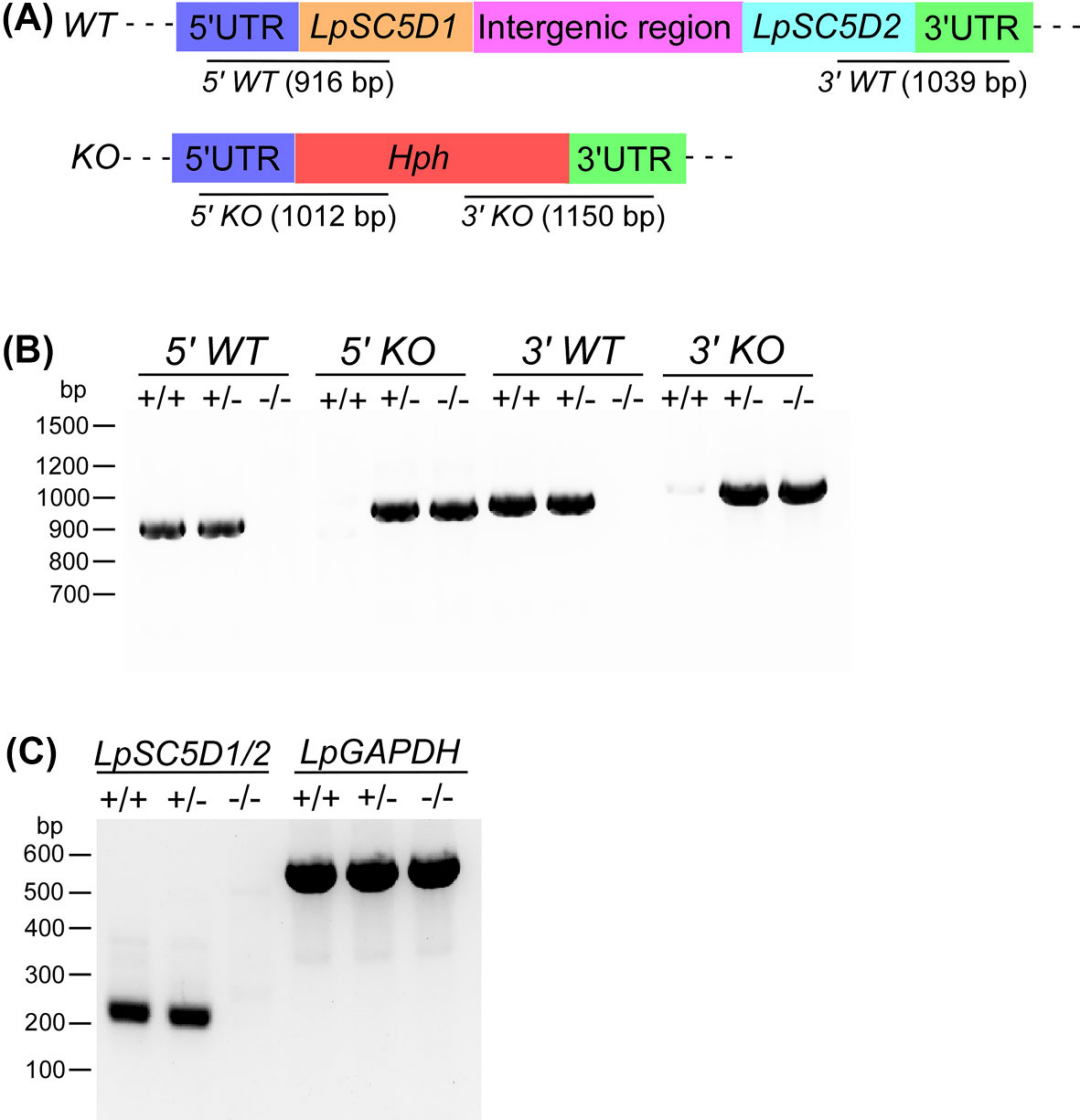
CRISPR-mediated deletion of *LpSC5D*. (A) Schematic of WT and deleted (KO) *LpSC5D* alleles generated by CRISPR/Cas9-induced homology-directed repair. The 5′ and 3′ UTRs, ORFs, intergenic region, and *Hph* are color-coded. Expected PCR product sizes for detecting 5′ WT, 3′ WT, 5′ KO, and 3′ KO alleles (not to scale) are shown. (B) Genomic DNA from WT (+/+), heterozygous (+/–), and homozygous (–/–) *LpSC5D* mutants was analyzed by PCR for the 5′ WT, 5′ KO, 3′ WT, and 3′ KO alleles. Molecular weight markers are indicated on the left. (C) RT-PCR detection of *LpSC5D1/2* (*LpSC5D1* and *LpSC5D2*) and *LpGAPDH* mRNAs in WT, heterozygous, and homozygous *LpSC5D* mutants using a forward primer specific to the *L. passim* splice leader sequence. Molecular weight markers are shown on the left.

As the four enzymes acquired by HGT participate in the early steps of ergosterol biosynthesis, their disruption is predicted to be lethal for *L. passim*. We thus targeted genes encoding sterol C-8 isomerase (LpSC8I) and sterol C-5 desaturase (LpSC5D), which act at the later stages of ergosterol synthesis (Fig. 1). The *L. passim* genome contains a single copy of *LpSC8I*, but two copies of *LpSC5D* (*LpSC5D1* and *LpSC5D2*). This duplication is also present in *L. pyrrhocoris, C. bombi*, and *Crithidia fasciculata*, but absent in *Leishmania* spp. and *Trypanosoma* spp. by TBLASTN analysis of the genomic contigs. These results suggest that *SC5D* duplicated in a common ancestor of monoxenous trypanosomatids. We found that LpSC8I, LpSC5D1, and LpSC5D2 co-localize with the ER marker protein LpBiP (Fig. 3), suggesting that these enzymes are concentrated in the ER. Thus, ergosterol biosynthesis in trypanosomatids likely occurs in both the glycosome and the ER. CRISPR-based homology-directed repair was used to disrupt *LpSC8I* (Liu et al. 2019). However, despite repeated attempts—including electroporation of a heterozygous knock-out clone with the knock-out construct—we failed to obtain homozygous mutants. This suggests that LpSC8I would be essential for viability in *L. passim*, and that Ergosta-8,24(28)-dienol can not substitute for ergosterol. We did not observe any growth or morphological phenotypes in the *LpSC8I* heterozygous knock-out clones. In contrast, we successfully disrupted both *LpSC5D1* and *LpSC5D2* by replacing their open reading frames with the hygromycin phosphotransferase (*Hph*) gene (Fig. 4A). Diagnostic PCR confirmed the absence of wild-type alleles at the 5' end of *LpSC5D1* and 3' end of *LpSC5D2* (Fig. 4B). RT-PCR using a reverse primer complementary to both *LpSC5D1* and *LpSC5D2* confirmed that both transcripts were absent in homozygous mutants (Fig. 4C). Unlike LpSC8I, LpSC5D is not essential for viability under culture conditions.

### Phenotypes of *LpSC5D*-deficient (*Lp*Δ*SC5D*) parasites

**Figure 5:**
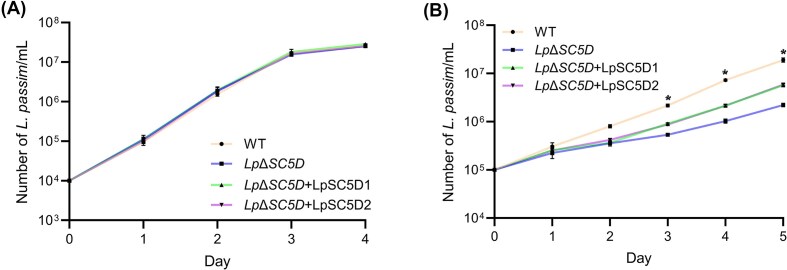
Growth of *LpSC5D*-deficient (*Lp*Δ*SC5D*) parasites in culture. (A) Growth of wild-type (WT; circles, beige line), *Lp*Δ*SC5D* (squares, blue line), *Lp*Δ*SC5D*+LpSC5D1 (triangles, green line), and *Lp*Δ*SC5D*+LpSC5D2 (inverted triangles, purple line) parasites at 30°C over 4 days (biological replicates, n = 3). (B) Growth of the same strains at 20°C over 5 days (biological replicates, n = 3). Statistically significant differences in growth between WT and *Lp*Δ*SC5D*, WT and *Lp*Δ*SC5D*+*LpSC5D1/2*, and *Lp*Δ*SC5D* vs. *Lp*Δ*SC5D*+*LpSC5D1/2* are indicated (asterisks; day 3: *P* < 0.001, *P* < 0.001, *P* < 0.005; day 4: *P* < 0.001, *P* < 0.001, *P* < 0.001; day 5: *P* < 0.001, *P* < 0.001, *P* < 0.02; Tukey HSD test).

**Figure 6:**
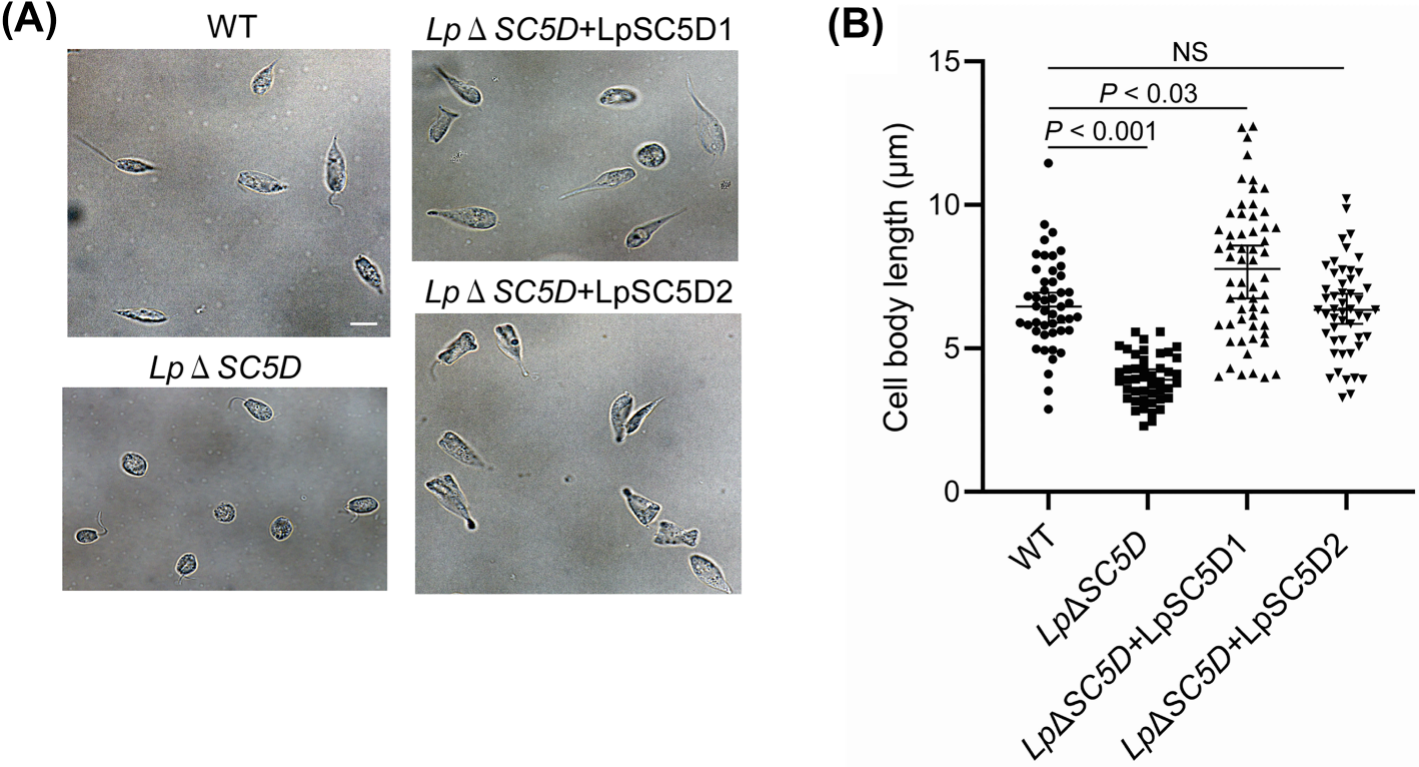
Morphology of *Lp*Δ*SC5D* parasites in culture at 20°C. (A) Morphology of WT, *Lp*Δ*SC5D*, and complemented parasites at day 4 during above 20°C culture. Scale bar: 5 µm. (B) Cell body length of WT (n = 47), *Lp*Δ*SC5D* (n = 45), *Lp*Δ*SC5D*+*LpSC5D1* (n = 58), and *Lp*Δ*SC5D*+*LpSC5D2* (n = 45). Data are shown as medians with 95% CI. Significant differences were found between WT and *Lp*Δ*SC5D* and between WT and *Lp*Δ*SC5D*+*LpSC5D1* (Steel test). NS: not significant.

**Figure 7:**
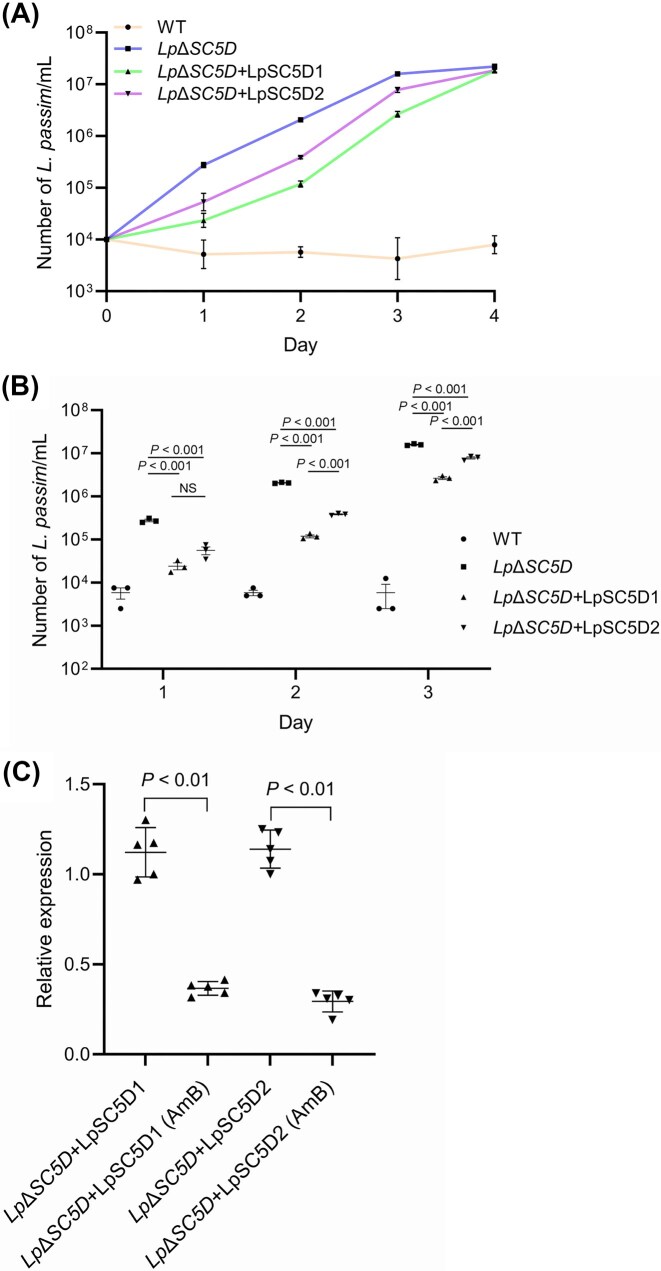
Growth of WT, *Lp*Δ*SC5D*, and complemented parasites under Amphotericin B (AmB).(A) Growth of WT, *Lp*Δ*SC5D*, and complemented parasites in the presence of 0.1 µg/ml AmB at 30°C over 4 days (biological replicates, n = 3). (B) Cell densities of WT, *Lp*Δ*SC5D, Lp*Δ*SC5D*+LpSC5D1, and *Lp*Δ*SC5D*+LpSC5D2 cultured with AmB across days 1–3. Mean values and SD are shown. Statistical significance was assessed using the Tukey HSD test. (C) Expression of *LpSC5D1* or *LpSC5D2* mRNA in complemented parasites with or without AmB treatment (biological replicates, n = 5). Relative mRNA abundance was calculated using the ΔCt method, with one untreated *Lp*Δ*SC5D*+LpSC5D1 or *Lp*Δ*SC5D*+LpSC5D2 sample set to 1. Statistical analysis was performed using the Mann–Whitney test.

To investigate the role of LpSC5D, we complemented mutants with plasmid-based expression constructs carrying either *LpSC5D1* or *LpSC5D2*, together with a bleomycin resistance marker (*Lp*Δ*SC5D*+LpSC5D1 or *Lp*Δ*SC5D*+LpSC5D2). Growth assays showed that while mutants grew normally at 30°C (Fig. 5A), they exhibited severely reduced growth under a low temperature stress condition at 20°C (Fig. 5B). Thus, ergosterol is required for efficient growth at low temperature. During mid-log growth at 20°C, wild-type parasites displayed elongated cell bodies with typical epimastigote morphology. In contrast, *Lp*Δ*SC5D* parasites had smaller, rounded cell bodies (Fig. 6A and B). Ectopic expression of either *LpSC5D1* or *LpSC5D2* rescued morphology but only partially growth (Figs 5B, 6A and B). As previously reported with *Leishmania* spp. and *L. passim* (Pourshafie et al. 2004, Pountain et al. 2019, Palmer-Young et al. 2022), wild-type *L. passim* was sensitive to Amphotericin B (AmB), with growth strongly inhibited at 0.1 µg/ml at 30°C (Fig. 7A). Amphotericin B binds strongly to ergosterol in the plasma membranes of fungi and trypanosomatids, inhibiting growth by forming membrane-disrupting pores. Intriguingly, *Lp*Δ*SC5D* parasites grew normally in the presence of AmB (Fig. 7A). Complemented strains (*Lp*Δ*SC5D*+LpSC5D1 and *Lp*Δ*SC5D*+LpSC5D2) grew more slowly in AmB than mutants, but eventually reached the same density after four days (Fig. 7A and B). We found that the expression of *LpSC5D1* and *LpSC5D2* mRNAs from the episomal plasmids was decreased in parasites cultured with AmB (Fig. 7C). This reduction likely contributes to their growth under AmB treatment. Notably, during the 2–3 days, *Lp*Δ*SC5D*+LpSC5D1 grew significantly more slowly than *Lp*Δ*SC5D*+LpSC5D2 (Fig. 7B).

### LpSC5D is required for conversion of ergosta-7,22-dienol to ergosterol

**Figure 8:**
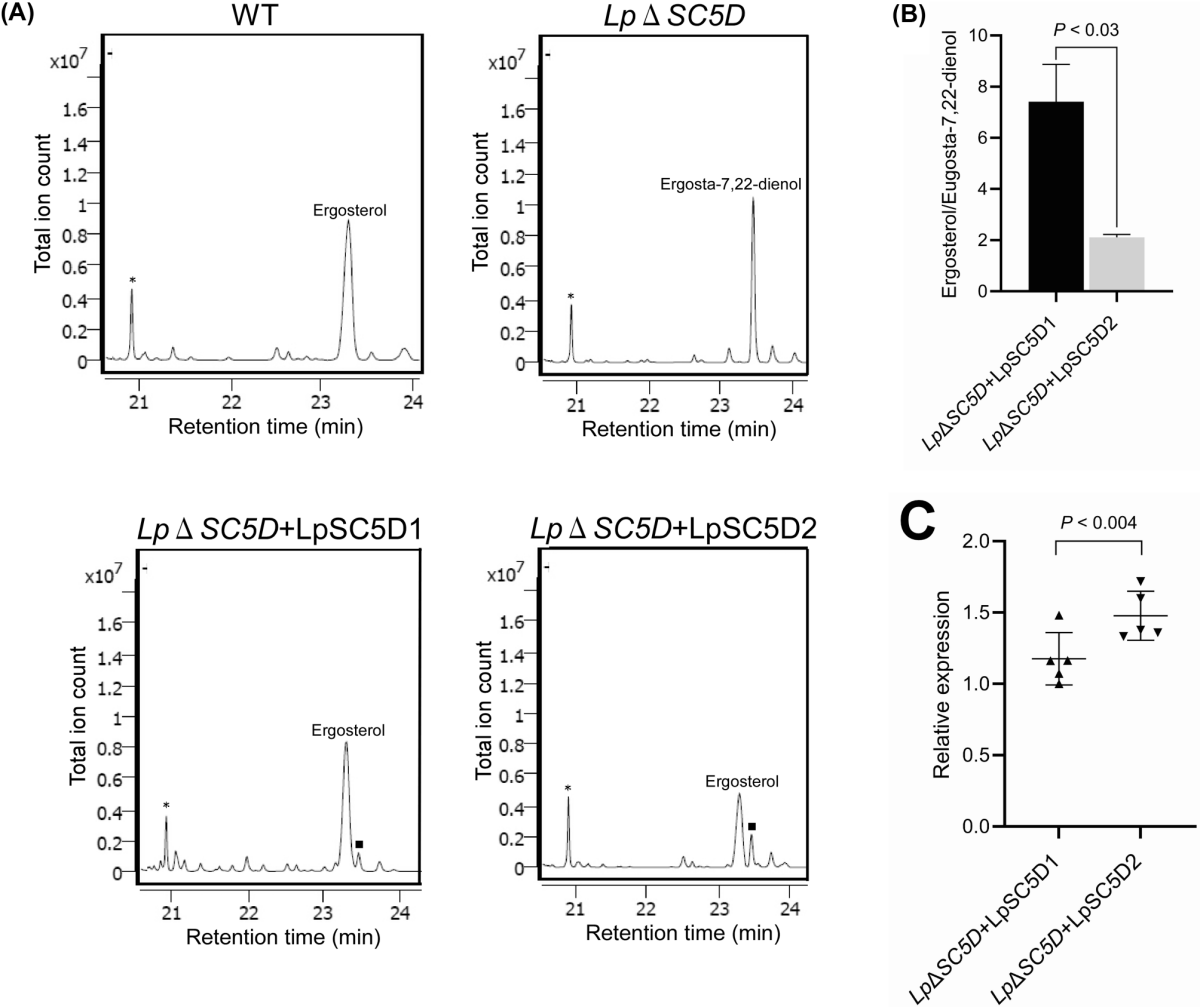
Accumulation of ergosta-7,22-dienol in *Lp*Δ*SC5D* and restoration of ergosterol synthesis in rescued parasites. (A) Partial GC-MS spectra of total sterols from WT, *Lp*Δ*SC5D, Lp*Δ*SC5D*+*LpSC5D1*, and *Lp*Δ*SC5D*+*LpSC5D2* parasites. Cholesta-3,5-diene (retention time = 20.9 min) was added as an internal standard (asterisks). Analyses were performed three times, and percentages of ergosterol (retention time = 23.3 min) and ergosta-7,22-dienol (retention time = 23.5 min) were quantified. Peaks corresponding to ergosta-7,22-dienol in *Lp*Δ*SC5D*+*LpSC5D1* and *Lp*Δ*SC5D*+*LpSC5D2* parasites are marked by squares. (B) Ratios of ergosterol to ergosta-7,22-dienol in *Lp*Δ*SC5D*+*LpSC5D1* and *Lp*Δ*SC5D*+*LpSC5D2* parasites (biological replicates, n = 3). Statistical analysis was performed using Welch’s *t*-test. (C) Expression of *LpSC5D1* and *LpSC5D2* mRNA in the complemented parasites (biological replicates, n = 5). Relative mRNA abundance was calculated using the ΔCt method, with one *Lp*Δ*SC5D*+LpSC5D1 sample set to 1. Statistical significance was assessed using the Brunner–Munzel test

GC-MS analysis revealed that wild-type *L. passim* produces ergosterol as its major sterol. In contrast, *Lp*Δ*SC5D* parasites predominantly accumulated ergosta-7,22-dienol (Fig. 8A), confirming that LpSC5D catalyzes its conversion to ergosterol (Fig. 1). Complemented parasites produced ergosterol as the major sterol, though significant amounts of ergosta-7,22-dienol remained. The ergosterol-to-ergosta-7,22-dienol ratio was higher in *Lp*Δ*SC5D*+LpSC5D1 than in *Lp*Δ*SC5D*+LpSC5D2 (Fig. 8B), consistent with the slower AmB growth of *Lp*Δ*SC5D*+LpSC5D1 (Fig. 7B). *LpSC5D2* mRNA was expressed at significantly higher levels than *LpSC5D1* mRNA in the complemented parasites (Fig. 8C). This suggests that LpSC5D1 has higher enzymatic activity than LpSC5D2.

### Ergosterol is essential for honey bee gut infection by *L. passim*

**Figure 9:**
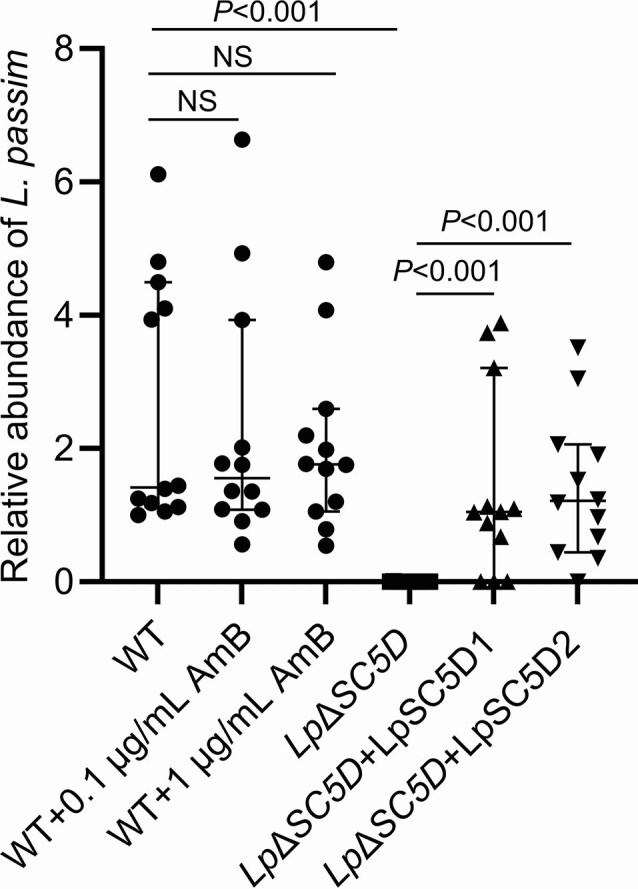
Infection of WT, *Lp*Δ*SC5D*, and complemented parasites in honey bee. Relative abundance of *L. passim* in honey bees (n = 12) 14 days post-infection with WT, WT fed 50% sucrose containing 0.1 or 1 µg/ml AmB, *Lp*Δ*SC5D, Lp*Δ*SC5D*+*LpSC5D1*, or *Lp*Δ*SC5D*+*LpSC5D2* parasites. Data were normalized to one WT-infected sample without AmB (set to 1) and are presented as medians with 95% CI. Statistical comparisons between WT and WT treated with 0.1 or 1 µg/ml AmB, as well as between WT and *Lp*Δ*SC5D*, were performed using the Steel test. Comparisons between *Lp*Δ*SC5D* and the complemented parasites were assessed using the Brunner–Munzel test.

We next tested infectivity in honey bees. Fourteen days after oral infection, *Lp*Δ*SC5D* parasites showed dramatically reduced colonization of the gut compared to wild-type parasites. The complemented parasites with either LpSC5D1 or LpSC5D2 showed the increased colonization (Fig. 9). This demonstrates that ergosterol is required for efficient honey bee gut infection. Feeding bees with sucrose containing 0.1 or 1 µg/ml AmB did not inhibit wild-type infection, likely due to drug inactivation in the gut environment (Fig. 9). *Leishmania mexicana* and *L. major* lacking sterol C14-α-demethylase (CYP51), sterol C24-methyltransferase (SMT1/2), or SC5D were previously generated (Xu et al. 2014, Mwenechanya et al. 2017, Mukherjee et al. 2019, Pountain et al. 2019, Ning et al. 2024); however, none of these mutants have been tested for infection competence in their sand fly vectors. These results demonstrate that ergosterol biosynthesis is critical for *L. passim* colonization of the honey bee gut.

## Discussion

This study clarifies the functional importance of ergosterol biosynthesis for the physiology and host association of *L. passim*, with particular emphasis on late-stage pathway enzymes and their contribution to growth, morphology, drug sensitivity, and infectivity. Although several enzymes involved in early sterol biosynthesis show atypical phylogenetic affiliations, the present findings indicate that parasite viability and host colonization are ultimately determined by the ability to synthesize ergosterol itself, rather than by the specific evolutionary origins of individual enzymes.

Our genetic analyses suggest a distinction between the essentiality of SC8I and SC5D in *L. passim*. The inability to generate homozygous *LpSC8I* knock-out mutants suggests that this enzyme appears indispensable for *L. passim* survival under culture conditions. This result implies that sterol intermediates upstream of SC8I, such as ergosta-8,24(28)-dienol, cannot functionally replace ergosterol in maintaining membrane integrity or associated cellular processes. In contrast, complete disruption of both LpSC5D paralogs was tolerated *in vitro*, indicating that the immediate precursor ergosta-7,22-dienol can partially substitute for ergosterol during growth at optimal temperature. Similar dispensability of SC5D has been reported in *Leishmania* spp. under culture conditions (Pountain et al. 2019, Ning et al. 2020, Alpizar-Sosa et al. 2022), underscoring a degree of flexibility in sterol composition that may buffer essential cellular functions in nutrient-rich environments.

Despite this apparent tolerance, the phenotypes of *Lp*Δ*SC5D* parasites reveal that ergosterol plays a critical role under physiologically relevant stresses. The pronounced growth defect at low temperature, accompanied by abnormal, rounded cell morphology, indicates that ergosterol is required to maintain membrane properties necessary for normal cell shape and proliferation under stress conditions. Temperature-dependent phenotypes are consistent with the well-established role of sterols in regulating membrane fluidity and phase behavior (Simons and Vaz 2004). In this context, ergosta-7,22-dienol appears insufficient to support membrane function when thermal conditions challenge lipid organization. The partial rescue of growth by episomal expression of either LpSC5D1 or LpSC5D2 further supports this interpretation.

Biochemical analyses confirmed that SC5D catalyzes the final conversion of ergosta-7,22-dienol to ergosterol in *L. passim*. The differential ergosterol-to-precursor ratios observed between LpSC5D1- and LpSC5D2-complemented lines suggest that the two enzymes are not functionally equivalent. Based on the lower expression of *LpSC5D1* mRNA from episomal construct, LpSC5D1 appears to possess higher catalytic activity than LpSC5D2. This functional divergence may explain the retention of both paralogs in several trypanosomatid lineages and could allow fine-tuning of sterol composition in response to environmental or developmental cues.

Altered sterol composition also had a marked impact on drug sensitivity. Loss of LpSC5D conferred resistance to amphotericin B, consistent with the known requirement of ergosterol for the pore-forming activity of this polyene antifungal (Gray et al. 2012). The gradual loss of amphotericin sensitivity in complemented lines under drug pressure is most parsimoniously explained by lower episomal LpSC5D expression, further reinforcing the direct link between ergosterol content and amphotericin susceptibility. These findings mirror observations in *Leishmania* spp. (Xu et al. 2014, Mwenechanya et al. 2017, Mukherjee et al. 2019, Pountain et al. 2019, Ning et al. 2020, Alpizar-Sosa et al. 2022, Jin et al. 2024, Ning et al. 2024, Tulloch et al. 2024) and highlight conserved sterol–drug interactions across trypanosomatids.

Most importantly, our infection experiments demonstrate that ergosterol biosynthesis is critical for successful colonization of the honey bee gut by *L. passim*. Although *Lp*Δ*SC5D* parasites remained viable in culture, their dramatically reduced infectivity indicates that ergosterol would be required for flagellar function (Povelones et al. 2023), survival, or proliferation, within the host environment. Conditions in the honey bee hindgut—such as anaerobic environment, variable nutrient availability, and exposure to host-derived stresses—likely exacerbate the functional limitations imposed by altered sterol composition. The failure of amphotericin B to suppress infection of wild type parasites *in vivo* further suggests that the gut environment diminishes drug activity, emphasizing that genetic disruption of sterol synthesis has far more profound effects on parasite infection than pharmacological inhibition under these conditions.

Taken together, our results establish ergosterol as a key determinant of *L. passim* fitness, particularly in the context of host infection. While certain sterol intermediates can sustain parasite growth *in vitro*, full ergosterol biosynthesis is required for maintaining normal morphology, environmental resilience, and infection competence. These findings underscore the central role of sterol metabolism in kinetoplastid biology and provide a framework for understanding how membrane composition shapes parasite adaptation to both culture and host-associated niches.

## Supplementary Material

xtag020_Supplemental_Files

## Data Availability

Taxonomy reference can be obtained from NCBI Taxonomy database (ftp://ftp.ncbi.nlm.nih.gov/pub/taxonomy). The NR database is available in NCBI FTP site (https://ftp.ncbi.nlm.nih.gov/blast/db/FASTA/). All other data is included in the manuscript and Supplementary material.
