# Supplementary material for: Role of ergosterol biosynthesis in growth, drug sensitivity, and host colonization of honey bee trypanosomatid parasite, Lotmaria passim
Source: FEMS Microbes. 2026 Apr 15;7:xtag020. doi: 10.1093/femsmc/xtag020 (PMC13142150; doi:10.1093/femsmc/xtag020)
Supplement: xtag020_Supplemental_Files [file xtag020_supplemental_files.zip › Supplementary Dataset 1.docx]

DNA sequences of *LpBiP*, *LpSC8I*, *LpSC5D1*, and *LpSC5D2*

>LpBiP

ATGGCAGTGAGGGATCGTCTTGTGCTGCTGGCGGTGTGCCTCGTGTCTGCACTGCTCATCGCAGCTGCGG

TCGCTGCACCGGACGGCAGTGGCAAGGTGGAGCCCCCGTGCATCGGCGTCGACCTCGGTACGACCTACTC

TGTCGCTGCCGTTTGGCAGAAGGGTGAGGTGCACATCATCACCAATGAAATGGGCAACCGCATCACCCCG

TCCGTTGTCGCCTTTACAGAGACGGAGCGGTTGGTCGGCGATGGTGCGAAGAACCAGCTCCCGCAGAACC

CGGAGAACACCATCTACGCCATCAAACGCCTGATTGGTCGTAAGTTCGCTGACCCAACGGTGCAGAACGA

CAAGAAGCTGCTGTCGTACAAGATTATCAGCGACAAGGCGGGCAAACCTCTCGTGCAGGTCACTGTGAAC

GGCGCCAAGAAGGAGTTCACGCCGGAGGAGGTGAGCGCGATGGTGCTGCAGAAAATGAAGGACATCTCGG

AGACCTTCCTTGGCGAGAAGGTGAAGAACGCCGTGGTGACGGTGCCCGCCTACTTCAACGACGCCCAGCG

CCAGGCGACGAAGGACGCCGGCAAGATTGCGGGCCTCAACGTGGTGCGCATCATCAATGAGCCGACGGCG

GCCGCCATCGCCTACGGCTTGAACAAGGCGGGGGAGAAGAACATCCTTGTCTTTGATCTGGGTGGCGGCA

CGTTCGATGTGTCGCTGCTGACCATCGACGAGGGCTTCTTTGAGGTGGTGGCGACGAACGGTGACACCCA

CCTCGGTGGCGAGGACTTCGATAACAGCATGATGAAGTTCTTCGTCGACGGCCTCAAGCGCAAGCAGAAC

ATCGATATTTCAAACGACCAGAAGGCGCTGGCGCGTCTGCGCAAGGCGTGCGAGGCGGCGAAGCGTCAGC

TCTCGTCGCACCCCGAGGCGCGTGTGGAGGTGGACAGCCTCGTAGAGGGCCACGACTTCAGCGAGAAGAT

TACGCGTGCCAAGTTTGAGGAGCTGAATATGGGCATGTTCAAGAACACCCTAATCCCGGTGCAGAAGGTG

CTGGAGGATGCGAAGCTGAAGAAGAGCGACATCGACGAGATCGTCCTCGTGGGTGGTTCGACCCGCATTC

CGAAAGTGCAGCAGCTGATCAAGGACTTCTTCGGCGGCAAGGAGCCGAACAAGGGCATCAACCCGGACGA

GGCCGTGGCGTATGGTGCGGCGGTGCAGGCGGCTGTGCTGATGGGTGAAAGCGAGGTCGGCGGCAAGGTC

GTTCTTGTTGATGTGATTCCCCTTTCCCTCGGCATCGAGACCGTCGGCGGCGTGATGACGAAGCTGATCG

AGCGCAACACGCAGATCCCGACCAAGAAGAGCCAGGTCTTCTCCACCTACCAGGACAACCAGCCCGGCGT

GCTCATCCAGGTTTTCGAAGGCGAACGGCAGATGACAAAGGACAATCGCCTACTAGGCAAGTTCGAGCTC

TCCGGCATCCCGCCGGCGCCGCGCGGAGTTCCACAGATCGAGGTCGCCTTCGACGTGGACGAGAACAGCA

TTCTGCAGGTGTCGGCGAGTGACAAGTCGTCTGGCAAGCGGGAGGAGATCACCATCACGAACGACAAGGG

TCGCCTGAGCGATGCGGAGATCCAGGCAATGGTGGAGGAAGCCGCGCAATTCGCTGAGGAGGACCGCAAG

GTGCGGGAGCGCGTGGAGGCGAAGAACTCGCTGGAGAGCATCGCGTACTCCCTGCGCAACCAGATCAACG

ACAAGGAGAAGCTTGGTGACAAGCTGGACGCGGACGATAAGAAGGCGATTGAGGCTGCCGTGCAGGTGGC

GCTCGATTTCGTCGACGAGAACCCGAACGCGGACCGCGAGGAGTTCGAGGAGGCGCGCGAGCAGCTGCAG

AAGGTAACGAATCCGATTATTCAGAAGGTGTACCAGGCTGCTGGCGGTGCCGCTGGTGAGGAGCCGGACG

CGATGGACGACTTGTAA

>LpSC8I

ATGCTCGGTTCTCGCTTCTCTGTGATGCTGGTAGCCCTCGTGGCGATCCTCATCGCGTTCTTCGTCTACGTGGACCAGCCCTCCAACTGGGTGTACGATCCGGCGCGCCTGCAGCAAATTGCGCAGCAGAGTATCGCCAACGCGAAGGCCGCCCATGGTGAGCACGCCACGGCAAAGCAGATCACCGACGAGACAATTCGGTTGATGCTCGAGGCGTACCCGCAGACGACGCGGAGCACCGGTCACTGGCTGTGGAACAACGCGGGCGGGGCGATGGGCTCCATGACGGTGCTGCACTGCTCCTTCTCGGAGTACATCATCATCTTCGGTACACCGGTCGGGACGGAGGGCCACACCGGGCGCTACTTCTGGGCCGAGGACTTCTTCAACATACTGGTGGGCGAGCAGTGGGCGGCGCTGCCTGGTGTGGCAGAGAGGGAGGTGTACCGCCCAGGTGATCAGCACGTCCTGCCGCGTGGCGTGGCGAAGCAGTACCGCATGCCGGACGAGTGCTGGGCTCTCGAGTATGCCCGTGGCAACATCGTCTCCATGCTCTTCTTTGGCTTCGCTGATATGCTGTCCTCGACGTTGGACGTGGTCACGACGTGGCATACGGTAGTGGAGAGTCTTGGCAACATGATCCCAAACCTGCTCGCTGGCAAGATTTAA

>LpSC5D1

ATGGACTTTGCCTTCAGGCTTTACGCCTCCGTCCTGCCGGTGGACAAGGACAAACTGACGCATCAGATGTTCATTTTTTGGCTGATCCTGACAACGGGTGGCACCTTCATGTACCTCTCCTTCGCTTCGCTGTCCTACAACATCTACTTCCGCCGCCTGAAGCAGCAGTTCTTCCCCAAGACGATCGACCCGGACAATACAACAGAACTACGACGGCAGGCCCTGCACGAGATCTGGATAGCGACGTGCTCCATCCCGTTCATGGCAGTACTGATGATGCCGGCTGCTGTCTTCTCGCACCGCGGCTACAGCAAAGTGTACTACAACATCTCCGATTACGGCTGGGCCTACTTCTTCCTCTCGCCCGTGCTGTTCTTCGCATTCACAGACTTCATGGTGTACTGCTTCCACCGTGGCTTGCATCACCCGATCATCTATAAGCACGTCCATAAGCTGCATCACACGTACAAGTTCACGACGCCCTTCTCCTCGCACGCCTTCAACCCGGTCGACGGGTTCGGTCAGGGTGTGCCGTACTACATCTTTGTTTATCTGTTCCCCCTGCACAATGTACTCTTCATGTGTCTTTTTGTGATGGTGAACTTCTGGACTATCTCGATTCACGACCAGGTGGACTTTGGCGGCCACTTTCTGAACACGACGGGCCACCACACGATCCACCACGAGCTGTTCAACTACGACTACGGGCAGTACACGACAGTGTGGGACCGCCTGGGTGGGACGTATCGTCCCGCGGAGCAGACGCACCAGATGACGACGCTGCTGCACGCGTGTGACCAGAATTACGTGGACCCTGTGTACGCGACGTACCACGACGAGAAGGGCTTCCTGGCTGGCCGCTTCAAGGAGGAGGCTGCAGCGCGTCACGTCAAGAAGGCTGCGTAG

>LpSC5D2

ATGGACTTTGCCTTTGACCTTTACACGTCTGTCCTGCCGGTGGACAAGGACAAACTGACGCATCAGATGTTCATTTTTTGGCTGATCCTGACAACGGGTGGCACCTTCATGTACCTCTCCTTCGCTTCGCTGTCCTACAACATCTACTTCCGCCGCCTGAAGCAGCAGTTCTTCCCCAAGACGATCGACCCGGACAATATGGCGGAGTTGTGGCGACAGGTCAAGCACGAGATCTGGATAGCGACGTGCTCCATCCCGTTCATGGCAGTACTGATGATGCCGGCTGCTGTCTTCTCGCACCGCGGCTACAGCAAAGTGTACTACAACATCTCCGATTACGGCTGGGCCTACTTCTTCCTCTCGCCCGTGCTGTTCTTCGCATTCACAGACTTCATGGTGTACTGCTTCCACCGTGGCTTGCATCACCCGATCATCTATAAGCACGTCCATAAGCTGCATCACACGTACAAGTTCACGACGCCCTTCTCCTCGCACGCCTTCAACCCGGTCGACGGCTTCGGTCAGGGTGTGCCGTACTTCATTTTCGTCTATCTTTTTCCTCTCCATCACCTTCTTTTCATCGCGCTCTTCATGTTGGTGAACTTCTGGACTATCTCGATTCACGACCAGGTGGACTTTGGCGGCCACTTTCTGAACACGACGGGCCACCACACGATCCACCACGAGCTGTTCAACTACGACTACGGGCAGTACACGACAGTGTGGGACCGCCTGGGTGGGACGTATCGTCCCGCGGAGCAGACGCACCAGATGACGACGCTGCTGCACGCGTGTGACCAGAATTACGTGGACCCTGTGTACGCGACGTACCACGACGAGAAGGGCTTCCTGGCTGGCCGCTTCAAGGAGGAGGCTGCAGCGCGTCACGTCAAGAAGGCTGCGTAG
