# Supplementary material for: Role of ergosterol biosynthesis in growth, drug sensitivity, and host colonization of honey bee trypanosomatid parasite, Lotmaria passim
Source: FEMS Microbes. 2026 Apr 15;7:xtag020. doi: 10.1093/femsmc/xtag020 (PMC13142150; doi:10.1093/femsmc/xtag020)

Supplementary dataset 2

Mass spectra of cholesta‑3,5‑diene (used as an internal control), ergosterol, and ergosta‑7,22‑dienol are shown.


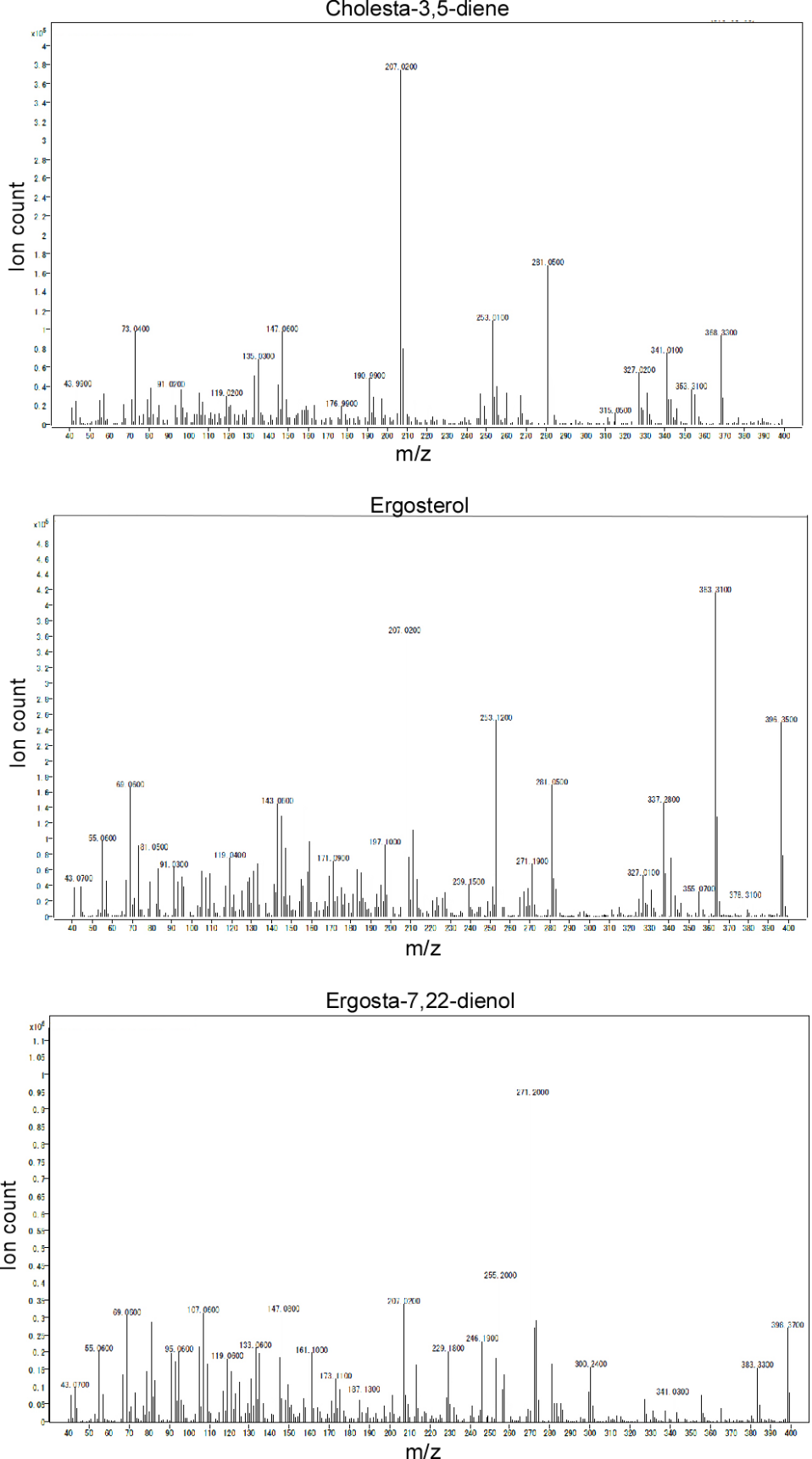

Supplement: xtag020_Supplemental_Files [file xtag020_supplemental_files.zip › Supplementary dataset 2.docx]
