# Supplementary material for: Role of ergosterol biosynthesis in growth, drug sensitivity, and host colonization of honey bee trypanosomatid parasite, Lotmaria passim
Source: FEMS Microbes. 2026 Apr 15;7:xtag020. doi: 10.1093/femsmc/xtag020 (PMC13142150; doi:10.1093/femsmc/xtag020)
Supplement: xtag020_Supplemental_Files [file xtag020_supplemental_files.zip › Supplementary dataset 3.docx]

Supplementary dataset 3

Amplification curves from the qPCR assays used to quantify WT and *Lp*Δ*SC5D* parasites in infected honey bees are shown. Amplification curves for the complemented lines (*Lp*Δ*SC5D* + LpSC5D1 or LpSC5D2) are also indicated. Am and Lp represent genome DNA of honey bee and parasite, respectively.

WT and *Lp*Δ*SC5D* parasites


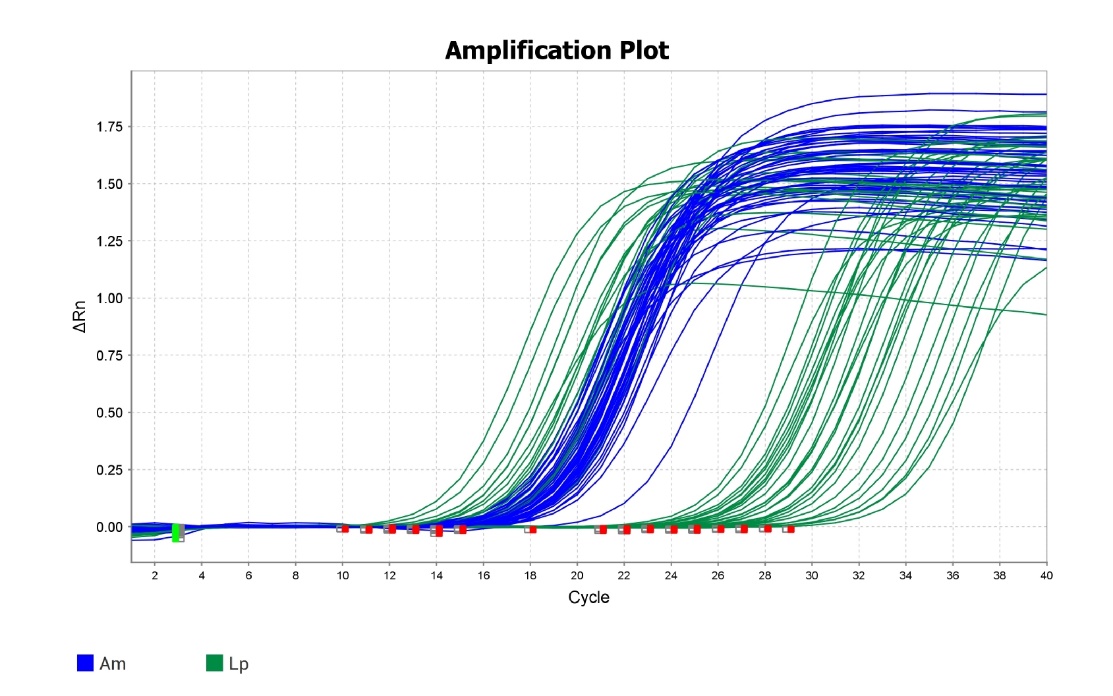


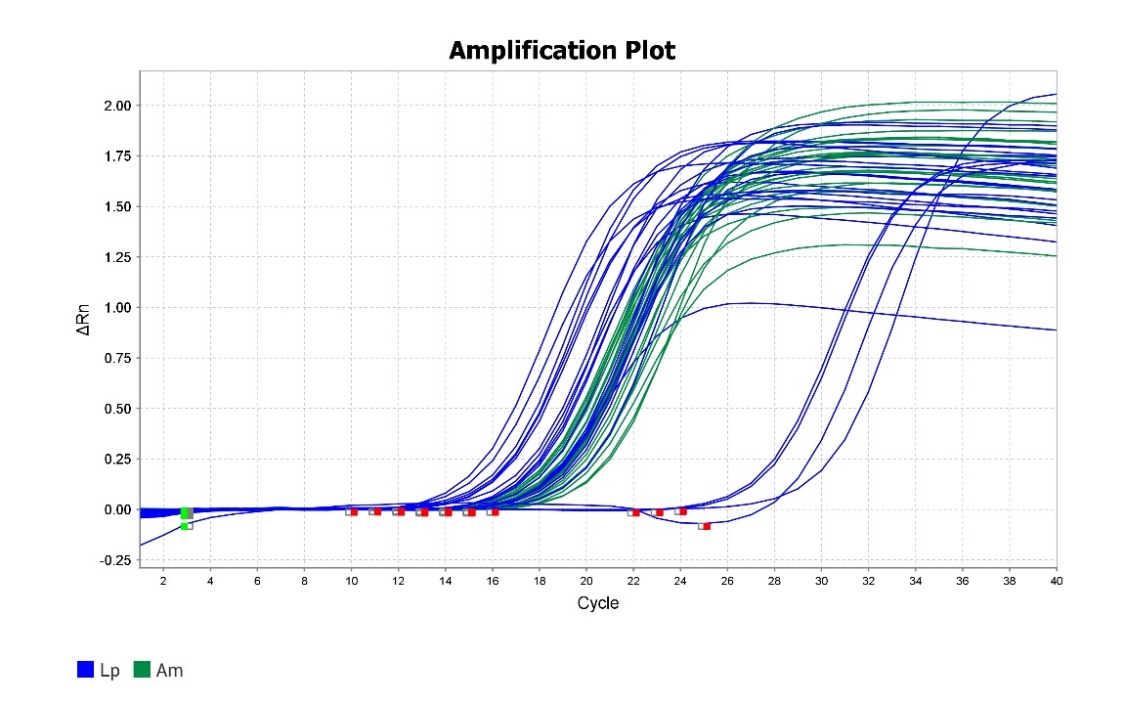
*Lp*Δ*SC5D* + LpSC5D1 or LpSC5D2 parasites
