# Supplementary material for: Role of ergosterol biosynthesis in growth, drug sensitivity, and host colonization of honey bee trypanosomatid parasite, Lotmaria passim
Source: FEMS Microbes. 2026 Apr 15;7:xtag020. doi: 10.1093/femsmc/xtag020 (PMC13142150; doi:10.1093/femsmc/xtag020)

Supplementary dataset 5

Phylogenetic trees of *Lotmaria passim* HMG‑CoA reductase, phosphomevalonate kinase, pyrophosphomevalonate decarboxylase, and farnesylpyrophosphate synthase (indicated in black) and their top 250 homologs inferred using the maximum‑likelihood method are shown. Notably, kinetoplastid proteins do not cluster with bacterial homologs.


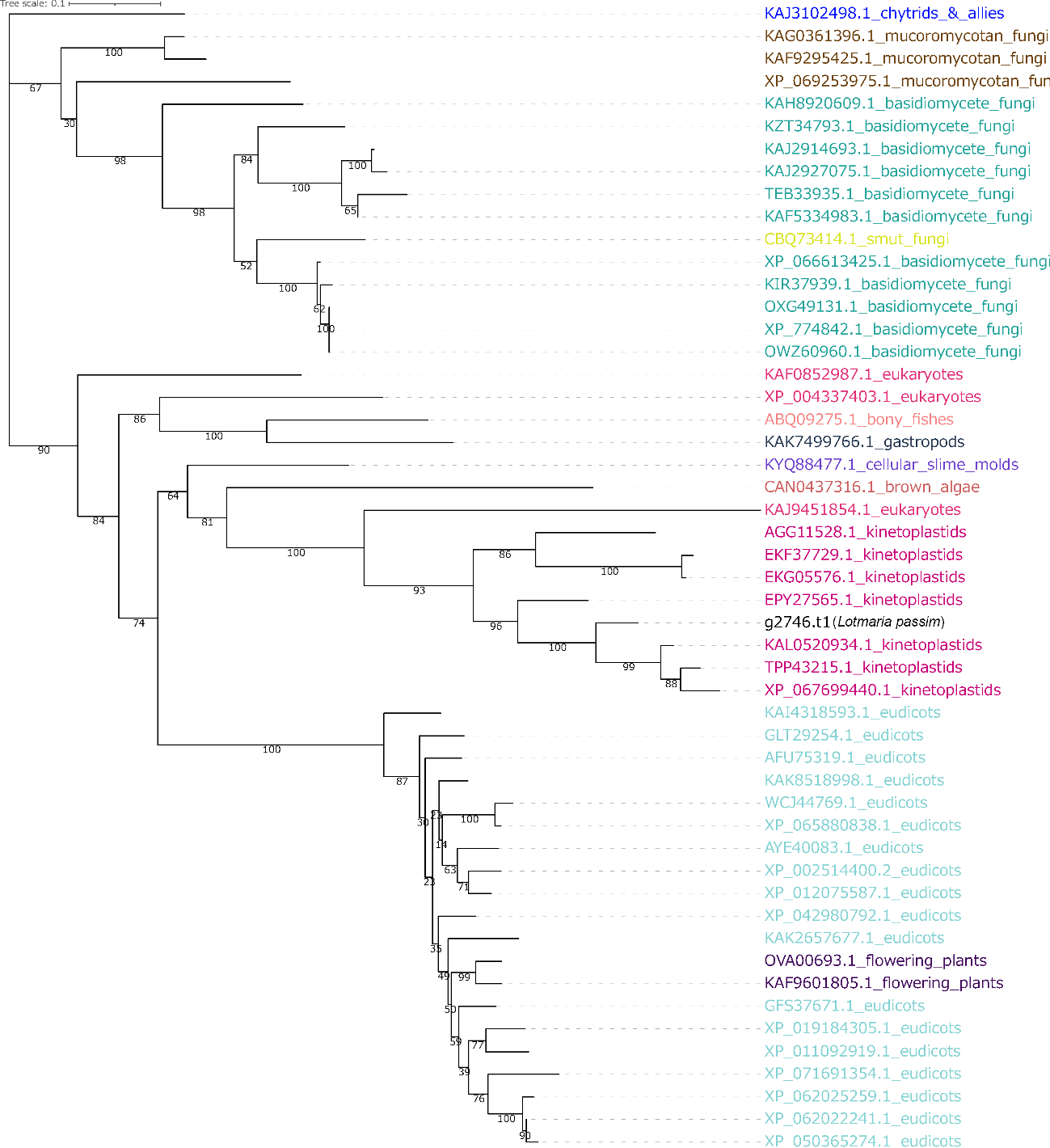


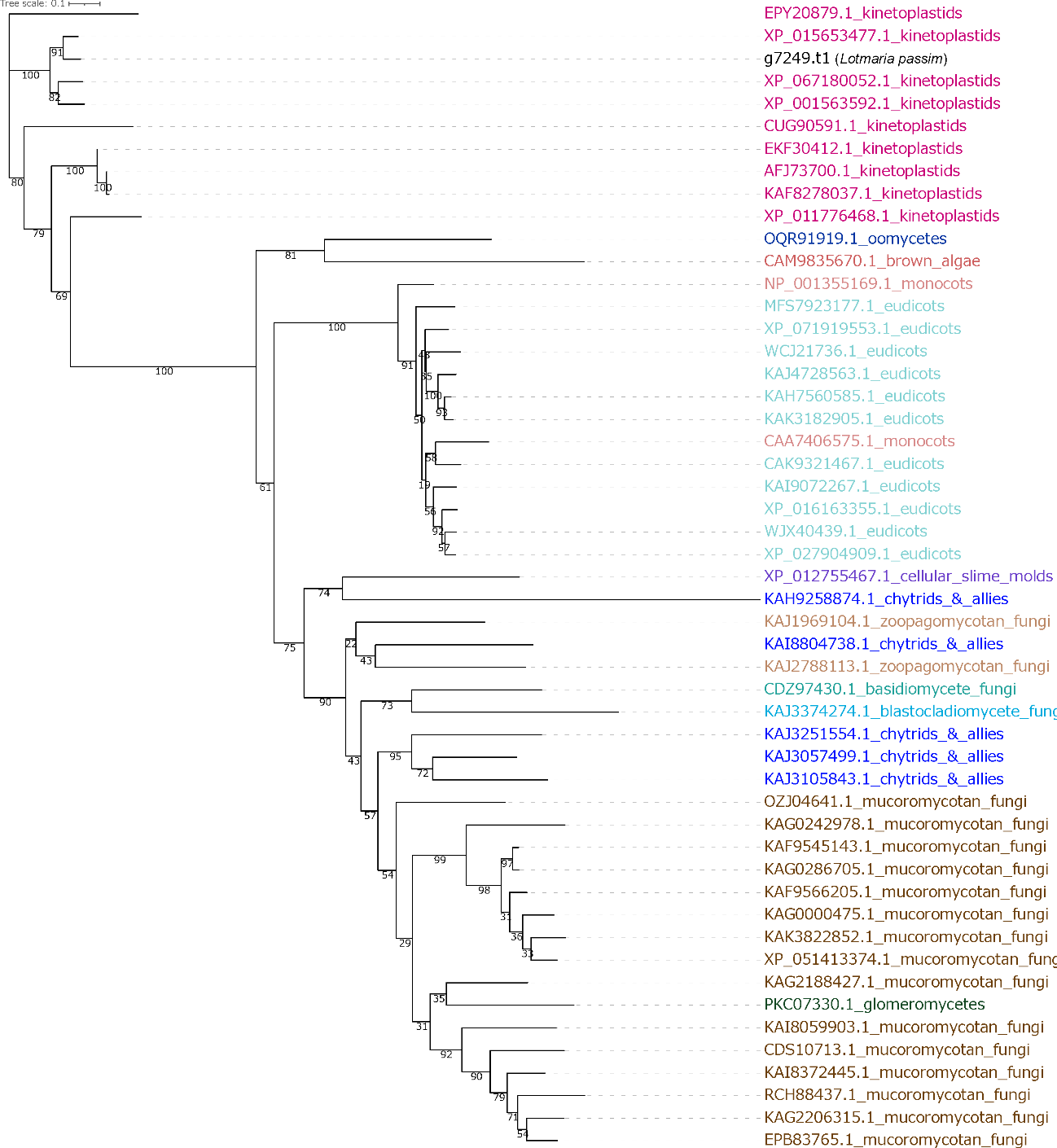


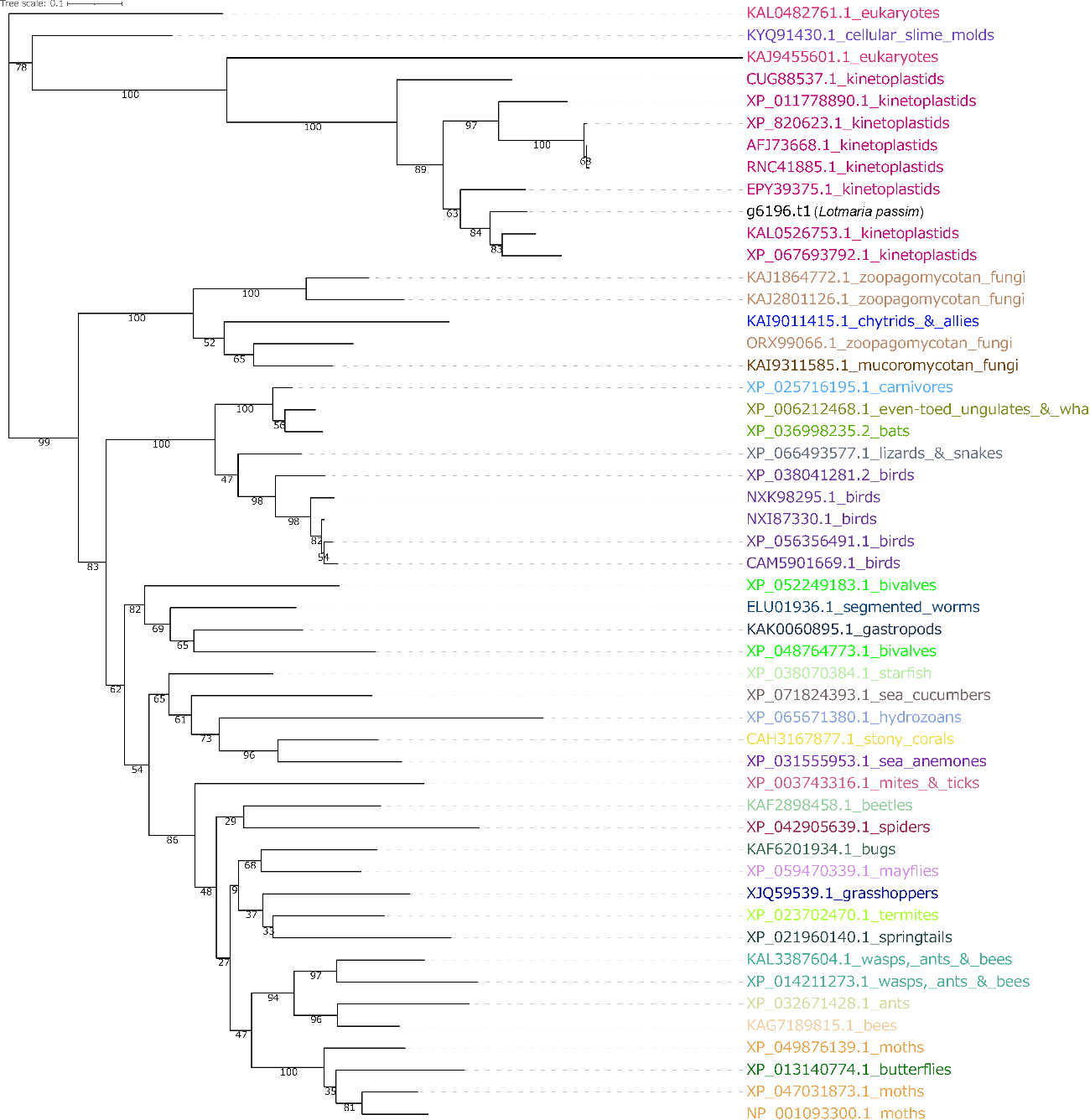


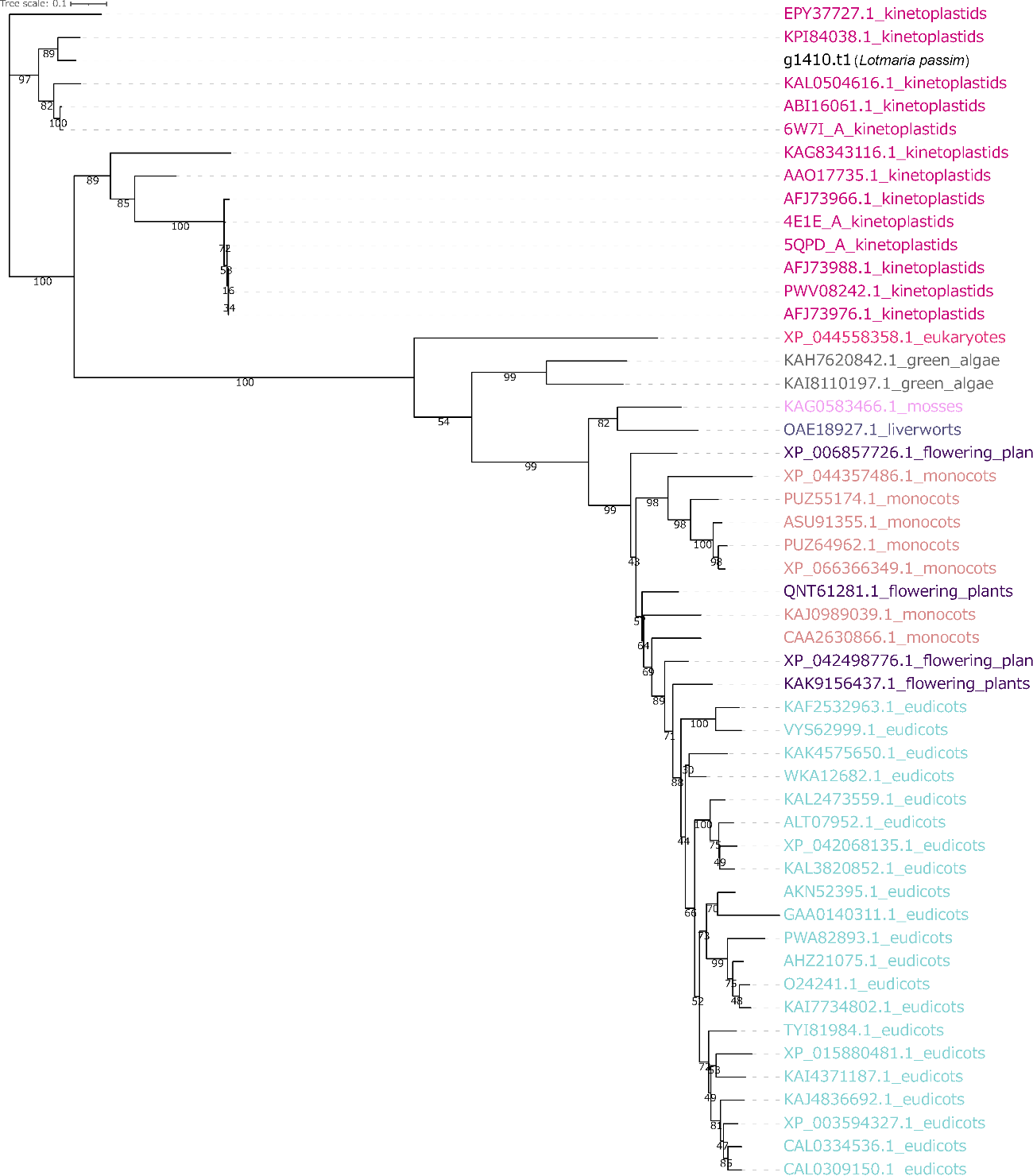

Supplement: xtag020_Supplemental_Files [file xtag020_supplemental_files.zip › Supplementary dataset 5.docx]
