# Supplementary material for: Role of ergosterol biosynthesis in growth, drug sensitivity, and host colonization of honey bee trypanosomatid parasite, Lotmaria passim
Source: FEMS Microbes. 2026 Apr 15;7:xtag020. doi: 10.1093/femsmc/xtag020 (PMC13142150; doi:10.1093/femsmc/xtag020)
Supplement: xtag020_Supplemental_Files [file xtag020_supplemental_files.zip › FEMSMC-2026-015.R1 supplementary data - reviews and author response.docx]

**Reviews of manuscript FEMSMC-2026-015.R1**

**Reviewer 1: Evan Palmer-Young, USDA**

**Recommendation: Minor Revision**

Yuan and Kadowaki report the results of an experimental manipulation of ergosterol synthesis in a trypanosomatid parasite of honey bees. They identify and perturb two enzymes of sterol biosynthesis, finding that transformants are inviable without function sterol C8 isomerase but can survive without a C5 desaturatase, although infection and cold tolerance are impaired in these ergosterol-deficient mutants.

As a trypanosomatid biologist, I find this study intriguing due to its combination of phylogenetics, biochemistry, and phenotypic analysis. It adds to the growing number of gene-disruption studies produced by this laboratory, with significance to the field of trypanosomatid biology as well as relevance to honey bee health with potential applied value.

The experiments are well done and adequately described, representing a coherent and impressively extensive series of manipulations requiring considerable effort and skill in molecular biology. The article is generally well-written and appropriate in the scope of the introduction and discussion.

I have mostly minor suggestions related to missing methodology and citations, as well as the presentation of the figures.

Line 75 'disruption of sterol synthesis can have diverse consequences'-- citations are needed here.

Methods: The genome and annotation used to identify the biosynthetic genes is not noted.

There is also no justification or citation given for which enzymes were chosen. Please clarify.

Line 111: 'homologs from diverse eukaryotic lineages'-- would be helpful to specify kingdom/phylum for these.

It is also somewhat confusing that the kinetoplastid Bodo saltans is not listed here, but its homologs are used for the phylogenies discussed.

Use of a second trypanosomatid (e.g. a Leishmania or Leptomonas) might also help to place the Lotmaria genes in the phylogeny relative to the much-discussed Leishmania.

Methods 2.2 Cellular localization. Defining acronyms at first use would be good form.

Line 255: Horizontal gene transfer "Consistent with prediction of glycosomal localization". Could authors add a few words of explanation? Are they referring to the evolutionary origins of the organelle from a formerly free-living organism?

Line 264: re: "duplication (of SC5D) is also present in" other monoxenous trypanosomatids. Some explanation should be given, here or in the Methods, of how this evidence was gathered (e.g., was it multiple BLAST hits to those genomes? A separate search of published annotations?)

Line 294: "wild-type L. passim was sensitive to Amphotericin". This was previously assayed in L. passim and monoxenous relatives in

Palmer-Young EC, Schwarz RS, Chen Y, Evans JD. Punch in the gut: Parasite tolerance of phytochemicals reflects host diet. Environmental Microbiology. 2022;24: 1805–1817. doi:10.1111/1462-2920.15981

Line 364: regarding the difference between SC5D1 and -D2, 'assuming comparable expression levels'-- is this something that could easily be checked by qPCR? For example, using RNA from the experiments in the figure with the gels? Although perhaps beyond the scope of the article, a biochemical characterization of the paralogous enzymes in vitro would be a bonus.

Line 387: "functional limitations imposed by altered sterol composition" for infection-- I suggest to also draw attention to the altered flagella, which are notable for involvement in epithelial attachment. A good citation is

Povelones ML, Holmes NA, Povelones M. A sticky situation: When trypanosomatids attach to insect tissues. PLOS Pathogens. 2023;19: e1011854. doi:10.1371/journal.ppat.1011854

Figure 1: Nice to see the full pathway. If this is from a public annotation, it would be helpful to see what gene numbers they correspond to. Another friendly touch would be to highlight, circle, or differently color the altered parts of the molecule in each step.

Figure 2: Would the phylogenies be clearer if they spelled out the genus names? I suggest to note in the figure legend that the red denotes a kinetoplastid lineage. Would it help to add a Leishmania species as well, since these species are referred to throughout the article?

The phylogenies for the other biosynthetic genes could also be shown somewhere for contrast, perhaps in a supplement?

Figure 3: This is a clever manipulation to show subcellular localization.

Fig 5: a lot is shown here!

I had a hard time distinguishing the lines on the graphs. Perhaps it would help to use larger shapes, different colors, or line types to differentiate them? Labeling the endpoints directly would also help a lot. I would suggest to be consistent across panels, mapping the same shapes/colors to the different lines.

Panel F would benefit from a legend and, if the authors like, might be switched to an interval plot (like in D and G) or line plot (like A, B) for consistency. Alternately, the growth curve data points in A and B could be horizontally offset like for the amphotericin growth curves. I leave aesthetics to the authors' discretion, so long as the distinctions between the lines are clear.

With seven(!) panels, the figure legend is lengthy, so it could also help to add a title above each graph to indicate which phenotype is being investigated (e.g., 20C growth, 30C growth, Amphotericin resistance, infection)

I hope these comments help the authors to polish their impressive contribution to the field.

**Reviewer 2**

**Recommendation: Major Revision**

Review on FEMSMC-2026-015_Proof_hi

The MS by Yuan and Kadowaki is one of the first approximations for understanding the biology of L. passim within honey bees. This makes this work interesting since this parasite is widely distributed and currently there few reports on the reasons why is so prevalent. Although technically complex (KOs, honey bee infections…) the MS still need to be work out for its acceptance.

General comments.

For easiness for the readers, please identify the KO cell lines with specific labels as (for instance Δ or KO) and the gene name in minorcase: e.g. change LpSDC5 by Lp Δ sdc5 or Lpscd5 KO all over the text and figures.

Graphical abstract: The parasites on the images are a bit too far. Could you at a higher magninfication of KO and WT parasites?

Abstract: I would avoid the word “transmission” since the experiments are focused to analyse thriving in the host. Transmission is not yet fully demonstrated in L. passim.

Introduction

Line 47: The order trypanosomatid which belongs to the Class Kinetoplastea is (or at least thus far) exclusively formed by parasite speces. Within the Class kinetoplastea is also composed by free-living organisms. Thus, please modify the “word” kinetoplastids” by Trypanosomatids.

Methods

Line 164, Change “was” by “where”.

Line 298 Have you tested new gRNAs instead of repeating transfections with same constructs?. What was your strategy for selecting gRNAs? gRNAs may vary in their efficiency (GC content, off targets, SNPs in the PAM region…etc)

Lines 192-202. This paragraph look like the description of your ectopically expressed proteins in KO cell lines (similarly to add-backs). So please generate a separate section from culture and cell body length or merged with the 2.3. Disruption of LpSC8I and LpSC5D genes by CRISPR section.

Line 207-208: Since the cell body of a promastigote is not completely round, Could you please describe more indeep how do you perform your cell body length in Image J?

Results

Linea 267. Please take carefully the potential loss in L. seymouri since the vast majority of those genomes are not chromosome-level resolved. Is it L. seymouri chromosome le el? If not please justify this sentence or delete It.

Figure 3. Could you please show a field with more than one parasite co-localising with Bip?

Line 283: Please justify the reason why you used Amphotericin B for phenotypic assay in ergsterol biosynthetic pathway and its relevance.

Line 313: In the section 3.5. Ergosterol is essential for honey bee gut infection by L. passim please provide a supplementary material the qPCR amplification curves.

Figure 5. In general, the differences between the lines representing the different groups of cell lines are difficult to distinguish. Please make the labels bigges (triangles, squares, etc) or do it with different line colours?

Figure 5B. Any rational reason why 20 C was proven for measuring parasite growth?? It is not really a physiological temperature within the host so results seem difficult to extrapolate…

Figure 5C and D. Could you please indicate the time point in the growth curve were the pictures where taken?

Figure 5F. Could you indicate in the figure a legend with the meaning of each of the colours?. Since this is a growth parameter, I would move it and as Figure 5C.

Discussion

Although experiments with bees always could bring difficulties, Experimental infections with rescued cell lines (add-backs) would have been ideal to test the effect observed by the deleted LpSC5D KO.

Line 372-375: To verify this statement, do a standard PCR with primers for the plasmid used for rescuing the parasite in Amp-treated cell line to test wether or not the plasmid still within the cells.

Line 383-384: Please delete attachment from this sentence since this is not either tested in vitro nor in vivo, or justify why attachment is included in the sentence

Supplementary dataset. Please provide the IDs of the genes and the genome used (NBI accession numbers) for isolating the sequences.

**Reviewer 3: Terry Smith, University of St. Andrews**

**Recommendation: Minor Revision**

The authors present a manuscript investigating the importance of ergosterol biosynthesis in Lotmaria passim. It is generally well written and well presented .

Major,

can the authors give a clear section on culturing L. passim, media conditions etc as I do not see this in the methods, the experimental strain used and its source?

Have the authors considered formation of a SKO of LpSC8I, may result in a phenotype

with out being lethal.

In figure 1 the authors have Acetyl-CoA going to directly to HMG-CoA via the HMG-CoA synthase, this is wrong , there is the acetyl-Coa acetyl transferase which makes acetoacetyl-CoA.

Some spectra of GC-MS peaks in supplementary would be nice

Minor

In several places the sub- or super-script seems to have extra "unnecessary" spaces associated

Ln 259 try and refrain from starting a sentence with "Because" , may be use "As"..

**Associate Editor Recommendation: Major Revision**

Thank you for your patience in waiting for the reviews. All reviewers appreciate the manuscript and have several constructive comments that you are requested to address in a revision.

**EIC Decision: Major Revision**

**Author's Response to Decision Letter for (FEMSMC-2026-015)**

Reviewer 1

Line 75 'disruption of sterol synthesis can have diverse consequences'-- citations are needed here.

Authors: The references have been added in the revised manuscript (Line 75-77).

Methods: The genome and annotation used to identify the biosynthetic genes is not noted.

There is also no justification or citation given for which enzymes were chosen. Please clarify.

Authors: We have added a new paragraph “2.2. Identification of L. passim genes involved in ergosterol biosynthesis” in the Materials and Methods section of revised manuscript (Line 108-127). This section now explains the genome annotation used and our approach for identifying L. passim genes involved in ergosterol synthesis. We also briefly describe the rationale for targeting LpSC8I and LpSC5D in the Results section (Lines 315–318).

Line 111: 'homologs from diverse eukaryotic lineages'-- would be helpful to specify kingdom/phylum for these.

It is also somewhat confusing that the kinetoplastid Bodo saltans is not listed here, but its homologs are used for the phylogenies discussed.

Use of a second trypanosomatid (e.g. a Leishmania or Leptomonas) might also help to place the Lotmaria genes in the phylogeny relative to the much-discussed Leishmania.

Authors: Kingdom and phylum information has been added for each species included in the phylogenetic analyses (Lines 133–145). We also clarified the identification of B. saltans, L. major, and T. cruzi orthologs (Lines 125–127, 145–147). Additionally, L. major or T. cruzi homologs were incorporated into the revised phylogenetic trees as recommended.

Methods 2.2 Cellular localization. Defining acronyms at first use would be good form.

Authors: The acronym has been defined in the revised manuscript (Line 157).

Line 255: Horizontal gene transfer "Consistent with prediction of glycosomal localization". Could authors add a few words of explanation? Are they referring to the evolutionary origins of the organelle from a formerly free-living organism?

Authors: The sentences have been revised to “These results suggest that multiple early enzymes in the sterol biosynthesis pathway were acquired by horizontal gene transfer (HGT) in the kinetoplastid ancestor, then recruited to glycosomes in trypanosomatid parasites. This is consistent with prediction of glycosomal localization of mevalonate kinase and isopentenyl-diphosphate delta-isomerase in L. major (Opperdoes and Szikora 2006)” (Line 305-310).

Line 264: re: "duplication (of SC5D) is also present in" other monoxenous trypanosomatids. Some explanation should be given, here or in the Methods, of how this evidence was gathered (e.g., was it multiple BLAST hits to those genomes? A separate search of published annotations?)

Authors: The text has been revised to “This duplication is also present in L. pyrrhocoris, C. bombi, and Crithidia fasciculata, but absent in Leishmania spp. and Trypanosoma spp. by TBLASTN analysis of the genomic contigs. These results suggest that SC5D duplicated in a common ancestor of monoxenous trypanosomatids.” (Line 320-323).

Line 294: "wild-type L. passim was sensitive to Amphotericin". This was previously assayed in L. passim and monoxenous relatives in

Palmer-Young EC, Schwarz RS, Chen Y, Evans JD. Punch in the gut: Parasite tolerance of phytochemicals reflects host diet. Environmental Microbiology. 2022;24: 1805–1817. doi:10.1111/1462-2920.15981

Authors: The sentence has been revised to “As previously reported with Leishmania spp. and L. passim (Pountain et al. 2019; Pourshafie et al. 2004; Palmer-Young et al. 2022), wild-type L. passim was sensitive to Amphotericin B (AmB), with growth strongly inhibited at 0.1 µg/mL at 30 °C (Fig. 7A).” (Line 352-355).

Line 364: regarding the difference between SC5D1 and -D2, 'assuming comparable expression levels'-- is this something that could easily be checked by qPCR? For example, using RNA from the experiments in the figure with the gels? Although perhaps beyond the scope of the article, a biochemical characterization of the paralogous enzymes in vitro would be a bonus.

Authors: We measured the mRNA levels of both genes in the complemented parasites and found that LpSC5D2 is expressed at significantly higher levels than LpSC5D1. The sentence has been revised to “LpSC5D2 mRNA was expressed at significantly higher levels than LpSC5D1 mRNA in the complemented parasites (Fig. 8C). This suggests that LpSC5D1 has higher enzymatic activity than LpSC5D2.” (Line 374-376). We plan to directly compare enzymatic activities in future work.

Line 387: "functional limitations imposed by altered sterol composition" for infection-- I suggest to also draw attention to the altered flagella, which are notable for involvement in epithelial attachment. A good citation is

Povelones ML, Holmes NA, Povelones M. A sticky situation: When trypanosomatids attach to insect tissues. PLOS Pathogens. 2023;19: e1011854. doi:10.1371/journal.ppat.1011854

Authors: The sentence has been revised to “Although LpΔSC5D parasites remained viable in culture, their dramatically reduced infectivity indicates that ergosterol would be required for flagellar function (Povelones, Holmes, and Povelones 2023), survival, or proliferation, within the host environment.” (Line 447-450)

Figure 1: Nice to see the full pathway. If this is from a public annotation, it would be helpful to see what gene numbers they correspond to. Another friendly touch would be to highlight, circle, or differently color the altered parts of the molecule in each step.

Authors: Enzyme Commission (EC) numbers have been added where applicable. We agree that some of the structural modifications are subtle so that they would be difficult to recognize. These alterations are highlighted with red in the revised Figure 1.

Figure 2: Would the phylogenies be clearer if they spelled out the genus names? I suggest to note in the figure legend that the red denotes a kinetoplastid lineage. Would it help to add a Leishmania species as well, since these species are referred to throughout the article?

The phylogenies for the other biosynthetic genes could also be shown somewhere for contrast, perhaps in a supplement?

Authors: Full species names are now shown in Figure 2, and L. major orthologs have been added. Because Acetyl-CoA-acetyltransferase is absent in Leishmania spp., T. cruzi was used instead. Bacteria with Mevalonate pathway contain enzymes up to Farnesyl pyrophosphate synthase. Thus, phylogenetic trees of HMG-CoA reductase, Phosphomevalonate kinase, Pyrophosphomevalonate decarboxylase, and Farnesyl pyrophosphate synthase are provided as Supplementary dataset 5. In these trees, L. passim protein and the top 250 homologs identified via NCBI BLASTP search against the non-redundant (nr) database are included. The kinetoplastid proteins do not cluster with the bacteria for the above four enzymes.

Figure 3: This is a clever manipulation to show subcellular localization.

Fig 5: a lot is shown here!

I had a hard time distinguishing the lines on the graphs. Perhaps it would help to use larger shapes, different colors, or line types to differentiate them? Labeling the endpoints directly would also help a lot. I would suggest to be consistent across panels, mapping the same shapes/colors to the different lines.

Panel F would benefit from a legend and, if the authors like, might be switched to an interval plot (like in D and G) or line plot (like A, B) for consistency. Alternately, the growth curve data points in A and B could be horizontally offset like for the amphotericin growth curves. I leave aesthetics to the authors' discretion, so long as the distinctions between the lines are clear.

With seven(!) panels, the figure legend is lengthy, so it could also help to add a title above each graph to indicate which phenotype is being investigated (e.g., 20C growth, 30C growth, Amphotericin resistance, infection)

Authors: The lines have been colored for clarity in the revised Figures 5 and 7. Panel F has been converted to a dot plot. The original Figure 5 has been divided into Figures 5–7 and 9.

Reviewer 2

General comments.

For easiness for the readers, please identify the KO cell lines with specific labels as (for instance Δ or KO) and the gene name in minorcase: e.g. change LpSDC5 by Lp Δ sdc5 or Lpscd5 KO all over the text and figures.

Authors: We have standardized the nomenclature throughout the manuscript and figures, using LpΔSC5D consistently in the revised version.

Graphical abstract: The parasites on the images are a bit too far. Could you at a higher magninfication of KO and WT parasites?

Authors: The images of both WT and KO parasites have been magnified in the revised graphical abstract.

Abstract: I would avoid the word “transmission” since the experiments are focused to analyse thriving in the host. Transmission is not yet fully demonstrated in L. passim.

Abstract: We have replaced “transmission” with “infection” in the revised Abstract (Line 40).

Introduction

Line 47: The order trypanosomatid which belongs to the Class Kinetoplastea is (or at least thus far) exclusively formed by parasite speces. Within the Class kinetoplastea is also composed by free-living organisms. Thus, please modify the “word” kinetoplastids” by Trypanosomatids.

Authors: We agree and have made this correction in the revised manuscript (Line 45).

Methods

Line 164, Change “was” by “where”.

Authors: We appreciate the suggestion; however, changing “was” to “where” would remove the verb from the sentence. Therefore, we retained the original wording (Line 196).

Line 298 Have you tested new gRNAs instead of repeating transfections with same constructs?. What was your strategy for selecting gRNAs? gRNAs may vary in their efficiency (GC content, off targets, SNPs in the PAM region…etc)

Authors: The gRNA sequences for LpSC8I and LpSC5D were designed using a custom gRNA design tool (http://grna.ctegd.uga.edu (grna.ctegd.uga.edu in Bing)) (Peng and Tarleton 2015). We selected gRNAs with the highest overall scores based on predicted on target and off target activity, nucleotide composition, and bracketing microhomology pairs. This information has been added to the revised manuscript (Lines 186–191). We did not test alternative gRNAs for LpSC8I because the successful generation of heterozygous KO clones indicated that the original gRNA was effective.

Lines 192-202. This paragraph look like the description of your ectopically expressed proteins in KO cell lines (similarly to add-backs). So please generate a separate section from culture and cell body length or merged with the 2.3. Disruption of LpSC8I and LpSC5D genes by CRISPR section.

Authors: The paragraph has been merged into section “2.5. Disruption of LpSC8I and LpSC5D genes by CRISPR” in the revised manuscript (Line 216-225).

Line 207-208: Since the cell body of a promastigote is not completely round, Could you please describe more indeep how do you perform your cell body length in Image J?

Authors: We now specify that the distance between the anterior and posterior ends of the cell body was measured (Lines 240–241).

Results

Linea 267. Please take carefully the potential loss in L. seymouri since the vast majority of those genomes are not chromosome-level resolved. Is it L. seymouri chromosome le el? If not please justify this sentence or delete It.

Authors: Because the L. seymouri genome assembly is of low quality, we removed the statement regarding the absence of SC5D duplication. However, we note that this duplication is absent in multiple Leishmania and Trypanosoma genomes with high quality assemblies (Lines 320–323).

Figure 3. Could you please show a field with more than one parasite co-localising with Bip?

Authors: Figure 3 has been revised to include images showing multiple parasites. Because both the GFP fusion protein and epitope tagged BiP are expressed episomally, expression levels vary among individual parasites, resulting in heterogeneous fluorescence in merged images.

Line 283: Please justify the reason why you used Amphotericin B for phenotypic assay in ergsterol biosynthetic pathway and its relevance.

Authors: Amphotericin B binds strongly to ergosterol in the plasma membrane of fungi and trypanosomatids, forming pores that inhibit growth. Previous work has shown that L. passim growth is repressed by Amphotericin B. This explanation has been added to the revised manuscript (Lines 352–357).

Line 313: In the section 3.5. Ergosterol is essential for honey bee gut infection by L. passim please provide a supplementary material the qPCR amplification curves.

Authors: We have added the amplification curves for WT, LpSC5D KO, and complemented KO parasites to Supplementary Dataset 3.

Figure 5. In general, the differences between the lines representing the different groups of cell lines are difficult to distinguish. Please make the labels bigges (triangles, squares, etc) or do it with different line colours?

Authors: All lines have been recolored for clarity in the revised Figures 5 and 7.

Figure 5B. Any rational reason why 20 C was proven for measuring parasite growth?? It is not really a physiological temperature within the host so results seem difficult to extrapolate…

Authors: We consider 20°C to represent a low temperature stress condition, and this rationale is now noted in the revised manuscript (Line 346).

Figure 5C and D. Could you please indicate the time point in the growth curve were the pictures where taken?

Authors: The images were taken on day 4 of the 5 day culture. This is now stated in both the Materials and Methods (Lines 240–241) and the figure legend (Lines 754–755).

Figure 5F. Could you indicate in the figure a legend with the meaning of each of the colours?. Since this is a growth parameter, I would move it and as Figure 5C.

Authors: Panel F has been converted to a dot plot in the revised Figure 7, as also suggested by Reviewer 1.

Discussion

Although experiments with bees always could bring difficulties, Experimental infections with rescued cell lines (add-backs) would have been ideal to test the effect observed by the deleted LpSC5D KO.

Authors: We agree, and these data have now been added as Figure 9 in the revised manuscript. The rescued parasites show more efficient host colonization than the parental KO parasites (Lines 380–381).

Line 372-375: To verify this statement, do a standard PCR with primers for the plasmid used for rescuing the parasite in Amp-treated cell line to test wether or not the plasmid still within the cells.

Authors: We examined the expression of LpSC5D1 and LpSC5D2 mRNA in rescued parasites cultured with Amphotericin B and found significantly reduced expression compared to parasites grown without the drug. This reduction may explain the continued growth of rescued parasites under drug pressure. These data are included in Figure 7 and described in the revised manuscript (Lines 360–362).

Line 383-384: Please delete attachment from this sentence since this is not either tested in vitro nor in vivo, or justify why attachment is included in the sentence

Authors: We have removed the word “attachment” as suggested (Lines 447–450).

Supplementary dataset. Please provide the IDs of the genes and the genome used (NBI accession numbers) for isolating the sequences.

Authors: We have not deposited the gene sequences in a database, but all sequences can be copied directly from the Supplementary Dataset. The accession number for the assembled L. passim genome used in this study is provided in the Materials and Methods (Line 110).

Reviewer 3

Major,

can the authors give a clear section on culturing L. passim, media conditions etc as I do not see this in the methods, the experimental strain used and its source?

Authors: A new section, 2.1. Culture of L. passim, has been added to the Materials and Methods (Lines 104–107).

Have the authors considered formation of a SKO of LpSC8I, may result in a phenotype

with out being lethal.

Authors: We successfully generated heterozygous KO clones of LpSC8I, as described in the manuscript. However, we did not observe any growth or morphological phenotypes. This information has been added to the revised text (Lines 332–333).

In figure 1 the authors have Acetyl-CoA going to directly to HMG-CoA via the HMG-CoA synthase, this is wrong , there is the acetyl-Coa acetyl transferase which makes acetoacetyl-CoA.

Authors: The Mevalonate pathway has been updated in Figure 1 to include Acetyl CoA acetyltransferase.

Some spectra of GC-MS peaks in supplementary would be nice

Authors: We have added mass spectra corresponding to Cholesta 3,5 diene, Ergosta 7,22 dienol, and Ergosterol in Supplementary Dataset 2.

Minor

In several places the sub- or super-script seems to have extra "unnecessary" spaces associated

Ln 259 try and refrain from starting a sentence with "Because" , may be use "As"..

Authors: These issues have been corrected throughout the revised manuscript.

**Reviews of manuscript FEMSMC-2026-015.R1**

**Reviewer 1: Evan Palmer-Young, USDA**

**Recommendation: Accept**

The authors have done excellent work addressing my comments.

The annotation and gleaning of enzyme sequences are better explained. The figure showing chemical reactions is really enhanced by the selective coloring of altered groups. The phylogenies are clarified using the extra kinetoplastids and full species names, and the clarity of the growth curves and dotplots are much improved. Additional quantifications of gene expression have been performed.

I am satisfied with the revisions.

In case it should be useful in the future, the cited Markowitz et al genome description of the Lp422 reference strain did include a complete annotation as a supplementary file, with considerably higher gene counts and BUSCO scores,

**Reviewer 3: Terry Smith, University of St. Andrews**

**Recommendation: Accept**

All of my concerns/ comments have been addressed

**Associate Editor Recommendation: Accept**

**EIC Decision: Accept**
